# Sphingosine-1-Phosphate Recruits Macrophages and Microglia and Induces a Pro-Tumorigenic Phenotype That Favors Glioma Progression

**DOI:** 10.3390/cancers15020479

**Published:** 2023-01-12

**Authors:** Lavinia Arseni, Rakesh Sharma, Norman Mack, Deepthi Nagalla, Sibylle Ohl, Thomas Hielscher, Mahak Singhal, Robert Pilz, Hellmut Augustin, Roger Sandhoff, Christel Herold-Mende, Björn Tews, Peter Lichter, Martina Seiffert

**Affiliations:** 1 Division of Molecular Genetics, German Cancer Research Center (DKFZ), 69120 Heidelberg, Germany; 2 Schaller Research Group at the University of Heidelberg and the German Cancer Research Center (DKFZ), 69120 Heidelberg, Germany; 3 Molecular Mechanisms of Tumor Invasion, German Cancer Research Center (DKFZ), 69120 Heidelberg, Germany; 4 Faculty of Biosciences, Heidelberg University, 69120 Heidelberg, Germany; 5 Division of Pediatric Neurooncology, German Cancer Research Center (DKFZ), Im Neuenheimer Feld 580, 69120 Heidelberg, Germany; 6 Division of Biostatistics, German Cancer Research Center (DKFZ), 69120 Heidelberg, Germany; 7 European Center for Angioscience (ECAS), Medical Faculty Mannheim, Heidelberg University, 68167 Mannheim, Germany; 8 Division of Vascular Oncology and Metastasis, German Cancer Research Center (DKFZ)-ZMBH Alliance, 69120 Heidelberg, Germany; 9 Lipid Pathobiochemistry, German Cancer Research Center (DKFZ), 69120 Heidelberg, Germany; 10 Department of Neurosurgery, Division of Experimental Neurosurgery, Heidelberg University Hospital, 69120 Heidelberg, Germany

**Keywords:** S1P, glioblastoma, microenvironment, tumor-associated macrophages/microglia, anti-inflammatory

## Abstract

**Simple Summary:**

A better understanding of the interactions between tumor and non-malignant cells is a priority to develop and improve therapeutic approaches for human glioma. Many lines of evidence have recognized sphingolipids as a family of lipids covering a wide range of functions in mammalian cells, in both physiological and pathological settings, including tumor–stroma crosstalk. In this study, we showed that higher amounts of sphingosine-1-phosphate (S1P) triggered an anti-inflammatory milieu both in vitro and in vivo. Inhibition of S1P signaling restored the anti-tumor response in vitro and improved the overall survival of mice that develop glioblastoma in vivo. The exploration of TCGA datasets allowed us to link high SPHK1 levels with a pro-tumorigenic phenotype in glioma patients, which ultimately resulted in a worse survival outcome. These results highlight the role of S1P in mediating tumor–stroma crosstalk, thus clarifying the power of S1P to be considered as a potential therapeutic target.

**Abstract:**

Glioblastoma is the most aggressive brain tumor in adults. Treatment failure is predominantly caused by its high invasiveness and its ability to induce a supportive microenvironment. As part of this, a major role for tumor-associated macrophages/microglia (TAMs) in glioblastoma development was recognized. Phospholipids are important players in various fundamental biological processes, including tumor–stroma crosstalk, and the bioactive lipid sphingosine-1-phosphate (S1P) has been linked to glioblastoma cell proliferation, invasion, and survival. Despite the urgent need for better therapeutic approaches, novel strategies targeting sphingolipids in glioblastoma are still poorly explored. Here, we showed that higher amounts of S1P secreted by glioma cells are responsible for an active recruitment of TAMs, mediated by S1P receptor (S1PR) signaling through the modulation of Rac1/RhoA. This resulted in increased infiltration of TAMs in the tumor, which, in turn, triggered their pro-tumorigenic phenotype through the inhibition of NFkB-mediated inflammation. Gene set enrichment analyses showed that such an anti-inflammatory microenvironment correlated with shorter survival of glioblastoma patients. Inhibition of S1P restored a pro-inflammatory phenotype in TAMs and resulted in increased survival of tumor-bearing mice. Taken together, our results establish a crucial role for S1P in fine-tuning the crosstalk between glioma and infiltrating TAMs, thus pointing to the S1P–S1PR axis as an attractive target for glioma treatment.

## 1. Introduction

Glioblastoma (GB) is the most common and aggressive malignant tumor of the central nervous system. It is generally associated with a poor prognosis and a median overall survival of 16 months from the time of diagnosis [1]. Therapeutic efficacy is profoundly hindered by several challenges such as tumor heterogeneity, altered cellular metabolism, and a unique immunosuppressive microenvironment. This consists of many different non-tumorigenic cells, including endothelial cells, pericytes, fibroblasts, and immune cells [2]. A unique feature of GB is the high abundance of myeloid cells. Indeed, tumor-associated macrophages/microglia (TAMs) define the largest population of tumor-infiltrating cells, representing one third of the total tumor mass [3]. In particular, this population is composed primarily of microglia and macrophages [3], either resident [4] or infiltrating from the circulation. TAMs are extremely plastic cells that, in the tumor, undergo morphological and phenotypical transition from a quiescent state to an activated one. Upon external stimuli, TAMs acquire a dynamic activation spectrum of which the most extreme phenotypes are known as so-called M1- or M2-type macrophages [5]. Although the M1/M2 nomenclature is an oversimplification of the actual complexity of the brain microenvironment, the M1 phenotype is functionally distinguished by its ability to eliminate pathogens and tumor cells, secrete pro-inflammatory cytokines, such as IL-23, IL-12, IL-6, IL-1β, and TNFα, and produce reactive oxygen species (ROS). On the other side, the M2 phenotype is characterized by the upregulation of several immunosuppressive factors, such as arginase 1, and by the secretion of anti-inflammatory cytokines, such as TGF-β and IL-10. In the tumor context, chemokines and growth factors secreted by glioma cells attract myeloid cells and promote their accumulation within the tumor tissue. Indeed, a direct correlation between the grade of gliomas and the amount of tumor-associated myeloid cells has been robustly demonstrated [3]. Nevertheless, the precise molecular mechanisms underlying these phenomena remain unclear.

Sphingosine 1-phosphate (S1P) is a biologically active lipid generated by the conversion of ceramide into sphingosine by the enzyme ceramidase, followed by the subsequent conversion of sphingosine into S1P, catalyzed by the enzyme sphingosine kinase (SPHK). S1P promotes cell survival and proliferation, whereas ceramide and sphingosine induce cell growth arrest and apoptosis [6]. These sphingolipids holding opposing functions are interconvertible inside cells, and their fine-tuned balance can determine cell fate. Sphingosine kinase 1 (SPHK1), one of the two identified SPHKs, regulates cancer progression, thus representing a critical player in the sphingosine-S1P balance. S1P binds to five G protein-coupled receptors, the S1P receptors (S1PR1–S1PR5), which are differentially distributed on distinct cell types. The heterodimerization of these receptors via different G-alpha subunits allows S1P to specifically exert its influence in numerous pathways to induce growth, differentiation, cell migration, and cell trafficking [7,8]. Enhanced S1P-driven signaling can contribute to a disease state and predispose to cancer onset [6]. Substantial evidence supports a deregulated production or degradation of S1P in the ‘hallmarks of cancer’ [6,9], and this was shown for a variety of cancers ranging from breast [10], ovarian [11], gastrointestinal [12], hepatocellular carcinoma [13], and glioblastoma [14]. Indeed, several of these studies show that increased SPHK1 expression correlates with higher tumor grade and shorter patient survival. Furthermore, the S1P–S1PR1 axis modulates immune responses in GB by sequestering T cells in the bone marrow [15]. In the present study, we demonstrate both in vitro and in xenogeneic glioma mouse models that the sphingolipid S1P secreted by glioma cells is able to recruit microglia and macrophages, thus promoting a tumor-supportive microenvironment. Moreover, our data suggest that the highly recruited TAMs are shifted towards an anti-inflammatory phenotype, which can be reverted by negatively modulating S1P. We also show that S1PR signaling is crucial to control the pro-inflammatory properties of TAMs. These observations are supported by our findings displaying a correlation between SPHK1 amounts and increased tumor-supporting TAMs in GB patients, thus pointing towards a pivotal role played by S1P in the glioma–microenvironment crosstalk and highlighting the possibility of targeting the SPHK–S1P–S1PR axis across these two compartments as a novel strategy in glioma therapy.

## 2. Materials and Methods

### 2.1. Cell Culture

All cells were cultured under humidified air conditions with 5% CO_2_ at 37 °C. Human LN229 and NCH82 GB cell lines, LN308 astrocytoma cell line, and the murine macrophage cell line RAW264.7 were grown in DMEM (Sigma Aldrich, Germany) containing 10% fetal calf serum (FCS-Biochrome, Germany) and 1% Penicillin-Streptomycin (Sigma Aldrich, Germany). HEK293 cells were cultivated in IMDM medium supplemented with 10% FCS, 1% Penicillin-Streptomycin [16]. After infection with lentivirus, cells were selected in growth medium containing 2–7 µg/mL of puromycin (BioMol, Germany). All cell lines were continuously monitored for mycoplasma according to the recommendations of the German Collection of Microorganisms and Cells (Germany). All cell lines were authenticated by Single Nucleotide Polymorphism (SNP)-profiling as described at www.multiplexion.de. Cell lines are listed in Supplementary Table S1. CYM-5442 (Tocris Biosciences, UK), W146, JTE-013, LPS, SKI-II (Sigma-Aldrich, Germany), Sphingosine 1-Phsophate, and SEW2871 (Cayman Chemical Company, USA) were used to treat murine primary microglia and RAW264.7 macrophages as specified in the figures and corresponding legends, with specific drug doses established based on previous literature [17,18] and on in-house cell viability tests.

### 2.2. PBMC Isolation and Monocyte Enrichment

Samples from heathy controls were obtained after informed consent and according to the guidelines of the Hospital Cliinic Ethics Committee, the Ethics Committee of the University of Heidelberg, and the Declaration of Helsinki. Peripheral blood mononuclear cells (PBMC) were isolated on a Ficoll density gradient (1.077 g/mL) by collecting the interphase cells after centrifugation at 1000× *g* for 20 min, as previously described [19]. Monocytes were isolated from PBMC using the Pan Monocyte Isolation Kit (130-093-537, Miltenyi Biotec, North Rhine-Westphalia, Germany) according to the manufacturer’s recommendation. Purity of monocytes was confirmed by flow cytometry staining with CD14 antibody prior to usage in experiments.

### 2.3. Animal Models

All animal procedures were conducted in accordance with the institutional animal research guidelines approved by the regional commission of Karlsruhe, Baden-Wuerttemberg, Germany (permit numbers G-287/15 and G-228/19). Athymic nude mice at the age of six to eight weeks were obtained from Envigo. LN308tRFP-2F-FLuc cells were harvested, washed in PBS, counted, and adjusted to 2 × 105 in 4 µL of PBS (AppliChem, Germany). Mice were anesthetized by intraperitoneal injection of 0.5 mg/kg body weight of medetomidin (Pfizer, Germany), 5.2 mg/kg body weight of midazolam (Ratiopharm, Germany), and 0.052 mg/kg body weight of fentanyl (Janssen, Germany). Mice were analgized by intraperitoneal injection of 4 mg/kg body weight of carprofen (Pfizer, Germany) 15 min before surgery. In addition, before starting the procedure, one drop of a lidocaine solution was dropped on the skull to ensure additional local analgesia. Mice were fixed in a stereotactic head frame (Kopf Instruments, USA). A 1 cm to 1.5 cm longitudinal incision was made and a hole was drilled through the skull (1 mm lateral and 2 mm posterior from the bregma). Either PBS or tumor cells were injected over 2 min at a depth of 2.5 mm using a 22-gauge syringe (Hamilton, Switzerland). After injection, the syringe was left in place for an additional 2 min and then slowly retracted. The incision was closed with stitches (Braun, Germany) and tissue glue (UHU, Germany). Mice were recovered from anesthesia by intraperitoneal injection of 2.6 mg/kg body weight of atipamezole (Pfizer, Germany), 0.5 mg/kg body weight of flumazenil (Roche, Germany), and 1.2 mg/kg body weight of naloxon (Inresa, Germany). SKI-II (MedChem) was solubilized in saline solution containing 2% DMSO, 30% PEG200, and 20% Tween80. Mice were treated with intraperitoneal injections of vehicle as control or SKI-II (50 mg/kg) three times a week, based on previous literature [20] and on dose and toxicity tests run in house.

### 2.4. Microglia Isolation

Murine microglia were isolated by mild trypsinization protocol as previously described [21]. Briefly, brains were removed from postnatal P0-P2 C57bl/6 pups and rinsed in Hanks Balanced Salt solution (HBSS, Sigma Aldrich, Germany). After removal of the meninges, the brains were mechanically dissociated and digested in 0.25% Trypsin (Sigma Aldrich, Germany) for 20 min. The cells were seeded in DMEM with 10% FCS and cultured under humidified air conditions with 5% CO_2_ at 37 °C. Medium was replaced every 4–5 days. Once they reached confluency, the mixed glial cultures were split 1:3 and expanded for an additional week in 10 cm cell culture dishes. Microglia were then isolated from the mixed glial cultures by incubation with 0.25% Trypsin-EDTA diluted 1:3 in serum-free DMEM for 45 min at 37 °C. After removal of the floating astrocyte layer, the adherent microglia were supplemented with mixed glial culture-conditioned media. After 24 h, 0.25% Trypsin-EDTA was added to the adherent microglia that were detached using a cell lifter (Corning Inc., USA). The cells were seeded in iBidi culture-insert (iBidi GmbH, Germany) at a density of 6 × 104 cells per well [16].

### 2.5. In Vitro Microglia-Glioma co-Culture

An in vitro co-culture model was established by using the Culture-insert 3-well in 35 mm μ-dish high (iBidi GmbH, Germany). The glioma cells were seeded in the area surrounding the 3-well inserts, and the primary microglia cells were seeded inside the inserts. After attaching, the cells were supplemented with fresh medium. A total of 48 h later, supernatants were collected and processed for ELISA assays and, simultaneously, RNA was isolated from primary microglial cells and analyzed for expression of M1 and M2 markers by real-time qPCR [16].

### 2.6. Chemoattraction Assays

24-well plates containing 5 µm pore size transwells were used for chemoattraction studies. Bottom chambers were filled with either 500 µL of complete medium or seeded with 25 × 103 glioma cell lines in DMEM containing 10% FBS and 1% Pen/Strep. After 24 h, fresh medium was added and allowed to condition for 48 h. Meanwhile, RAW264.7 macrophages were serum starved for 12–16 h and 6 × 104 seeded on the transwells in serum-free DMEM medium. Transwells were incubated at 37 °C for 6 h. For the assays with human monocytes, the cells were freshly isolated from human PBMC and 5 × 104 seeded on the transwells in serum-free DMEM medium. Transwells were incubated at 37 °C for 3 h. Post migration, the transwells were washed in PBS and the cells present on the upper side of the transwells were removed with the help of a cotton swab. Cells on the lower side of the transwell membranes were fixed with PBS (AppliChem, Germany) containing 4% formaldehyde (Roth, Germany) overnight at 4 °C and stained with 4′,6-diamidino-2-phenylindole (DAPI, Sigma Aldrich, Germany). Cells were imaged using a Cell Observer microscope (Zeiss, Germany), recorded using ZEN 2012 software (Zeiss, Germany), and counted using ImageJ v1.47 (National Institutes of Health, USA).

### 2.7. Plasmid Construction

Plasmid construction was performed as previously described [22]. Plasmids are listed in Supplementary Table S2.

### 2.8. Lentivirus Production

Lentivirus production was performed as previously described [22]. shRNA primers are listed in Supplementary Table S3.

### 2.9. Bioluminescence Imaging

All mice were imaged at day 7, 14, and 21 post tumor cell injection. D-luciferin was injected at a concentration of 150 mg/kg body weight (Biomol, Germany). Mice were anaesthetized using 1.5% isoflurane (Abbott, Germany). After 10 min incubation, images were acquired using an IVIS Lumina II system (Caliper Life Science, USA) with exposure times of 1, 3, and 5 min. Bioluminescence signals were quantified with LivingImage 4.4 (PerkinElmer, USA).

### 2.10. Flow Cytometry and Cell Sorting

For brain isolation, mice were anesthetized with 1.5% isoflurane (Abbott, Germany) and transcardially perfused with PBS and 4% paraformaldehyde (PFA). Mouse brain specimens were macrodissected and dissociated using the Neural Tissue dissociation kit (NTDK, Miltenyi) and a single-cell suspension generated using the GentleMACS dissociator. Tissue suspensions were filtered through a 70-μM mesh filter and underwent red blood cell lysis (Pharm-Lyse BD) and myelin removal with Myelin Removal Beads (Miltenyi). Single-cell suspensions were blocked by incubation for 15 min at 4 °C in 2% rat serum and then stained with cell-surface antibodies and fixable viability dye for 30 min at 4 °C. Cells were subsequently washed twice with FACS buffer and sorted in PBS with 5% FCS using BD FACSAria II (BD Biosciences, USA) running with FACSDiva Software (BD Biosciences). Cells were then centrifuged and resuspended in RNA lysis buffer (Qiagen) before snap freezing in liquid nitrogen and storage at –80 °C. Data were analyzed with FlowJo X 10.0.7 software (FlowJo, Ashland, OR, USA).

### 2.11. Trizol-Based RNA Isolation

RNA isolation from primary microglia was performed using Trizol. Briefly, cells were washed with PBS, and 800 μL of PeqGoldTriFast reagent (PEQLAB Biotechnologie) was added to the plate and incubated at RT for 5 min. Cells were collected and transferred in a 1.5-mL tube. 160 μL of chloroform was added, vortexed thoroughly, incubated at RT for 2–3 min, and centrifuged at 13,000× *g* for 15 min at 4 °C. The upper aqueous phase containing RNA was transferred into a new tube without disturbing the intermediate organic layer. Following this, 500 μL of ice-cold isopropanol and 1 μL of RNA-free glycogen (PEQLAB Biotechnologie) per 200 μL of aqueous layer were added, vortexed briefly, and incubated at −20 °C overnight. The following day, the solution was centrifuged at 13,000× *g* for 2 h at 4 °C. The supernatant was discarded, and the pellet was washed with 1 mL cold 70% ethanol and then air-dried at RT and subsequently dissolved in DEPC-free water. RNA quality and concentration was measured by NanoDrop spectrophotometer (Peqlab, Erlangen, Germany) [16].

### 2.12. Quantitative Reverse Transcription Polymerase Chain Reaction (qRT-PCR)

Total RNA was isolated from cells using the RNeasy Mini Kit (Qiagen, Hilden, Germany) and reverse transcribed using the First Strand cDNA Synthesis Kit (Thermo Scientific, Germany). Transcripts were quantified on a LightCycler 480 (Roche, Germany) using the FastStart SYBR Green Master Kit (Roche, Germany). Fluorescence was recorded and analyzed using the LightCycler Gene Scanning Software 1.5 (Roche, Germany). Relative quantification of target genes was done against reference genes using the 2-ΔΔCt or the 2-ΔCt method. Primers are listed in Supplementary Table S4.

### 2.13. Active Rac1/RhoA Pull-Down Assay

LN229 cells were seeded in 15-cm dishes at a density of 1.5 × 106 cells and incubated at 37 °C o.n. The following day, the plates were washed in PBS and fresh DMEM containing 10% FBS and PS was added and allowed to condition for 48 h. Meanwhile, RAW264.7 cells were cultured up to 60–70% confluence in 15-cm dishes and serum starved for 24 h. The media conditioned by the tumor cells were then collected, briefly centrifuged to remove cells, and added on the starved RAW264.7 for the indicated time points. RAW264.7 were then lysed on ice with 700 µL of lysis buffer. Levels of active Rac1/RhoA were measured using the RhoA/Rac1 Activation Assay Kit (Cytoskeleton, USA) according to manufacturer’s instructions. Briefly, cells were lysed in 3× lysis buffer containing 150 mM Tris, pH 7.5; 30 mM MgCl2; 1.5 M NaCl, and 6% Igepal and the insoluble material was removed by centrifugation at 10,000 rcf for 1 min at 4 °C. Clarified lysates were incubated overnight at 4 °C with 15 µg of PAK-PBD beads for active Rac1 pull-down and 50 µg of Rhotekin-RBD beads for active RhoA pull-down. Beads were then washed once with washing buffer (25 mM Tris, pH 7.5; 30 mM MgCl2; 40 mM NaCl), resuspended in 40 µL of Laemmli buffer, and boiled for 5 min. Western blots using 4–20% gradient gels were performed for Rac1 or RhoA to quantify Rac1/RhoA precipitation for each condition.

### 2.14. Immunoblotting

Immunoblot of total lysates was performed as previously described [22]. Antibodies are listed in Supplementary Table S5. Original WB can be found in Figure S6.

### 2.15. Enzyme-Linked Immunosorbent Assay (ELISA)

IL-10, TNFα, and IL-6 were quantified using BioLegend Mouse ELISA MAX Standard kit (BioLegend, San Diego, CA, USA) according to the manufacturer’s protocol. The cellular supernatant of microglia cultures treated with glioma-conditioned media was harvested and briefly centrifuged to remove floating cells. The 96-well ELISA plates were washed twice in PBS and coated overnight at 4 °C with 100 μL of Capture antibody. Wells were washed and blocked with assay diluent for 1 h at room temperature on a plate shaker. Plates were washed and 100 μL/well of standard dilutions and 100 μL/well of appropriate sample dilutions were added and incubated on a plate shaker at 500 rpm for 2 h at RT. The plate was washed and incubated with 100 μL of Detection antibody on a plate shaker at 500 rpm for 1 h at RT. The plate was washed and incubated with 100 μL of Avidin-HRP solution on a plate shaker at 500 rpm for 30 min at RT. Finally, the plate was washed and incubated with 100 μL of tetramethylbenzidine (TMB) substrate solution in the dark for 15–30 min at RT. The reaction was stopped by adding 100 μL of Stop solution and absorbance was read at 450 nm within 15 min with the iMark microplate absorbance reader (BioRad, Germany). The absolute values of cytokines levels were calculated from a standard curve by plotting the standard cytokine concentration and the absorbance [16].

### 2.16. Griess Reagent Assay

The amount of nitrite in cell lysates was measured through the Griess reagent assay (Sigma-Aldrich, St. Louis, MO, USA) according to manufacturers’ instructions. Briefly, cells were resuspended in ice cold Nitrite Assay Buffer and chilled on ice for 10 min. Samples were centrifuged at 10,000× *g* at 4 °C for 5 min and the supernatants transferred to fresh tubes. A total of 10–100 μL of sample per well was added and the volume was adjusted to 100 μL with Nitrite Assay Buffer. Nitrite levels were then detected by measuring absorbance at 540 nm with an iMark microplate absorbance reader (BioRad, Germany).

### 2.17. S1P Determination by UPLC-ESI-(QqQ)-MS2

To determine S1P content, samples were transferred into screw-capped glass vials (SCGV) and evaporated to dryness under constant nitrogen stream at 39 °C. As an internal standard, 27.4 pmol d17:1-S1P was added per sample. A total of 2 mL chloroform/methanol (1:2) and 0.1% trifluoroacetic acid (*v*/*v*) were added, followed by vigorous vortexing. Subsequently, 1 mL chloroform and 1 mL ddH2O were added. Samples were vortexed after each addition. Phase separation was facilitated by centrifugation at 3500× *g* for 15 min. The lower phase was collected in a new SCGV. The upper phase was reextracted with 1 mL chloroform and pooled with the first extract. The extracts were dried under constant nitrogen stream at 39 °C. After resuspension in 100 µL methanol, lipid extracts were transferred to GC vials. S1P was derivatized by adding 10 µL trimethylsilyldiazomethane (2M in hexane) from a freshly prepared aliquot. The derivatization reaction proceeded for 20 min at room temperature under gentle agitation and was stopped by addition of 1 µL acetic acid. Samples were dried and reconstituted in 150 µL methanol:water (95:5) for subsequent analysis. Sphingosine-1-phosphate derivatives were analyzed by UPLC-ESI-(QqQ)-MS2 (Waters Acquity I-class ultra-performance LC with Xevo TQ-S triple quadrupole mass spectrometer). Measurements were done in reversed-phase-LC mode using a UPLC CSH C18 column (2.1 × 100 mm, 1.7 µm; Waters) at a flow rate of 0.4 mL/min with the gradient indicated in Supplementary Table S6. Multiple reaction monitoring (MRM) mode was used to monitor specific transitions for 4x-methylated S1P(d17:1) and 4x-methylated S1P(d18:1) (Supplementary Table S7).

### 2.18. Human Glioma Datasets

Normalized and log2-transformed microarray gene expression data (Affymetrix U133A) of the TCGA-GB cohort were downloaded using the TCGAbiolinks package. Association of gene expression with SPHK1 expression levels was assessed with limma [23]. Enrichment of gene sets in terms of association with SPHK1 expression levels was analyzed with the camera test [24] and displayed with barcode plots. Samples were clustered using Euclidean distance and Ward’s linkage. The Kruskal–Wallis test was used to compare expression levels between subtypes.

RNA-Seq read count data of the TCGA-GB cohort (N = 166) were downloaded using the TCGAbiolinks package. Association of gene expression with SPHK1 expression levels was assessed with edgeR [25]. Enrichment of gene sets in terms of association with SPHK1 expression levels was analyzed with the camera test [4] and displayed with barcode plots. For display in heatmaps, Fragments Per Kilobase Million (FPKM) values were used and log2-transformed. Samples were clustered using Euclidean distance and Ward’s linkage. The Kruskal–Wallis test was used to compare expression levels between subtypes.

### 2.19. Survival Analysis

Patients of the TCGA cohorts were grouped into high or low gene expression groups applying the maximal selected ranked statistics method [26,27]. Survival difference between the groups was estimated by fitting an age-adjusted cox proportional hazard model. Kaplan–Meier curves were used for visualizing the fitted cox proportional hazard models. The adjusted *p* values of the maximally-selected log rank statistic for the cut-point search are shown [28].

### 2.20. Statistical Analysis

Graphical and statistical analyses of qRT-PCR and protein were performed using GraphPad Prism software version 7. Unless otherwise indicated, data represent the mean ± SEM of at least three independent experiments. Depending on the data, the following statistical analyses were applied: two-tailed Student’s *t* test (paired or unpaired), one-way ANOVA with Tukey’s multiple comparisons test, and repeated-measures ANOVA with Dunn’s multiple comparisons test. The statistical details including the statistical test used for each dataset are indicated in the figure legends. Significant differences were reported as * *p* < 0.05, ** *p* < 0.01, *** *p* < 0.001, and **** *p* < 0.0001. n.s. indicates no significant difference.

Statistical analysis of mouse data was performed using GraphPad Prism software version 7. All graphs show means ± SEM, unless otherwise indicated. Comparisons of two different sample groups were performed using a two-tailed unpaired Student’s *t* test with Welch approximation to account for unequal variances. The statistical details including the statistical test used for each dataset are indicated in the figure legends. Significant differences were reported as * *p* < 0.05, ** *p* < 0.01, and *** *p* < 0.001. n.s. indicates no significant difference.

## 3. Results

### 3.1. High SPHK1 Expression Correlates with Poor Survival and High Microglia Recruitment in GB

**Figure 1:**
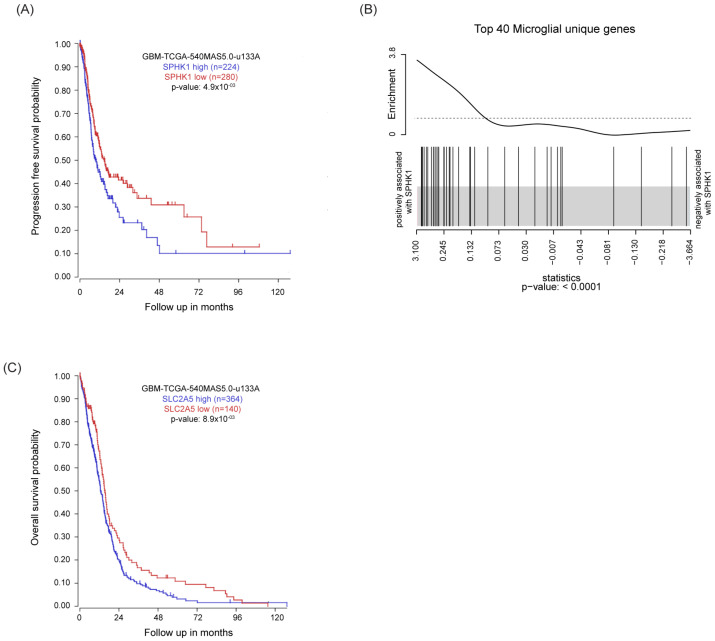
**High SPHK1 expression correlates with poor survival and high microglia recruitment in GB.** (**A**) Kaplan–Meier plot estimates progression-free survival in patients with glioblastoma divided into high and low SPHK1 expression. (**B**) Gene set enrichment analysis (GSEA) of microglia gene signature defined in Butovsky et al., 2014 [30] on TCGA RNAseq data of the GB dataset. The barcode plot shows the positive association between SPHK1 mRNA expression level and the top 40 microglial-unique genes. (**C**) Kaplan–Meier plot estimates progression-free survival in patients with glioblastoma divided into high and low SLC2A5 expression. KM analyzed using the Affymetrix 540 MASS 5.0-u133 array available in the online R2: Genomics Analysis and Visualization Platform (http://r2.amc.nl, accessed on 10 October 2022). *p* value in Kaplan–Meier plot results from scan modus test.

SPHK1 is often upregulated in cancer, and high levels of SPHK1 have been associated with tumor invasion, angiogenesis, and resistance to radio- and chemotherapy [29]. Following up on these observations, we analyzed survival of GB patients in relation to SPHK1 expression using the TCGA GB dataset and revealed that high SPHK1 is associated with worse outcome both in terms of progression-free survival and overall survival (Figure 1A and Figure S1A). Among the various factors contributing to tumor progression, the tumor microenvironment (TME) strongly influences tumor invasion and treatment response. By analyzing the relation between SPHK1 expression in GB and gene signatures of different cell types within the TME [30], we showed that SPHK1 was positively associated with the microglial gene signature (Figure 1B), in particular with the expression of genes such as CSF1R [31], SLC2A5 [32], TMEM119 [33], CX3CR1 [34], and P2RY12 [35] (Figure S1B), but less associated with astrocyte, oligodendrocyte, and neuron gene signatures. Interestingly, higher expression of the above-mentioned genes correlated with a poor survival outcome in the microarray GB dataset. We report one exemplary graph for gene SLC2A5 (Figure 1C), which also nicely correlated with SPHK1 expression in the whole transcriptome GB dataset (Figure S1C). Altogether, these results suggest that SPHK1 is strongly interconnected with the microglia compartment in GB, and that its enzymatic product S1P could therefore play an important role in the crosstalk between the tumor-associated macrophages/microglia (TAMs) and glioma cells.

### 3.2. S1P Acts as a Chemoattractant for Murine Macrophages via the Modulation of Rac1/RhoA

**Figure 2:**
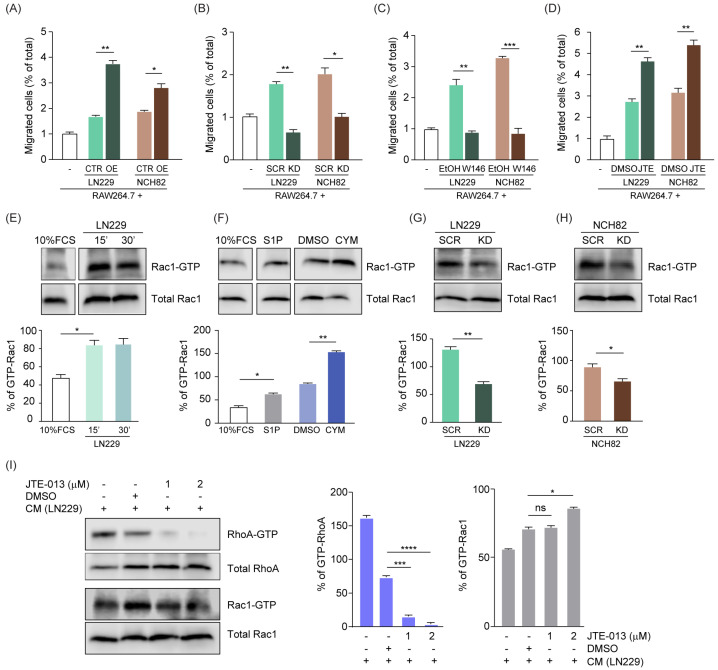
**S1P acts as a chemo-attractant for murine macrophages via the modulation of Rac1/RhoA.** (**A**,**B**) Chemoattraction assay of murine RAW264.7 macrophages towards human glioma cells LN229 and NCH82, either overexpressing (OE, (**A**)) or with knockdown (KD, (**B**)) of SPHK1 versus corresponding control cells (CTR and SCR, respectively). (**C**,**D**) Chemoattraction assay of RAW264.7 macrophages towards LN229 and NCH82, treated with either W146 (**C**) or JTE-013 (**D**) and corresponding solvent controls (EtOH or DMSO, respectively). Data are expressed as percent increased or decreased migration in a time frame of 6 h. (**E**–**H**) Rac1-GTP pull-down in RAW264.7 macrophages treated with media conditioned by LN229 cells for the indicated times, (**E**) 1 μM S1P, 500 nM CYM-5442, or DMSO control for 15 min (**F**). Media conditioned by LN229 (**G**) or NCH82 (**H**) cells with knockdown for SPHK1 (KD) versus the corresponding scrambled siRNA controls (SCR) for 15 min. (**I**) RAW264.7 cells pretreated with JTE-013 at the indicated concentrations or with corresponding solvent control and then exposed to media conditioned by LN229 cells for 15 min. Cell lysates were harvested and analyzed for active RhoA and Rac1 levels via pull-down assay. In (**E**–**I**), the percentage of active Rac1-GTP and active RhoA-GTP was calculated based on band quantification using ImageJ, and normalizing Rac1-GTP against total Rac1 or RhoA-GTP against total RhoA, respectively. CYM-5442 (S1PR1 agonist). Means with SEM are shown, n = 3, representative data are shown. ns: not significant, * *p* < 0.05, ** *p* < 0.01, *** *p* < 0.001, **** *p* < 0.0001 by Kruskal-Wallis test for (**A**–**F**,**I**), and by unpaired *t*-test for (**G**,**H**).

In order to investigate the effect of S1P on microglia and macrophages, we modulated S1P levels by generating stable cell lines with either overexpression or knockdown for SPHK1. We first evaluated the efficiency of this modulation in human cell lines representative of GB (LN229, NCH82) or astrocytoma (LN308) both at the transcript (Figure S2A,B) and protein level (Figure S2C–E). Furthermore, we quantified S1P by LC/MS-MS and confirmed increased amounts of S1P in cells overexpressing SPHK1, and reduced levels of the phospholipid in SPHK1-downregulated cells (Figure S2F). These cell lines were then used in chemoattraction assays in combination with RAW264.7 murine macrophages. We evaluated the percentage of RAW264.7 cells migrating over a 6-h incubation time across the Boyden chamber and observed that the GB cell lines triggered macrophage migration, which was significantly increased with SPHK1-overexpressing cells (Figure 2A) and significantly reduced with SPHK1-knockdown cells (Figure 2B) compared to the corresponding control cells. These results corroborate the intimate interaction between SPHK1 and S1P and point towards a crucial role for S1P as a chemoattractant for macrophages.

Among the five different G-protein coupled receptors (S1PR1–5) that bind S1P, sphingosine-1-phosphate receptor 1 (S1PR1) and 2 (S1PR2) are clearly linked to macrophage recruitment and act in opposite ways, with S1PR1 promoting and S1PR2 blocking cells attraction in response to S1P [36]. Therefore, we modulated S1PR1 and S1PR2 in macrophages and then evaluated their migration rate over 6 h in the presence of the parental LN229 or NCH82 cells. We observed a reduction of macrophage migration up to 3-fold (Figure 2C) when blocking S1PR1 via W146 [37], whereas the inhibition of S1PR2 by JTE-013 [17] significantly increased the S1P-mediated macrophage chemoattraction (Figure 2D).

We then extended this approach to human monocytes freshly isolated from peripheral blood mononuclear cells (PBMC) and observed that they are more strongly recruited by the parental LN229 cells (Figure S2G) and exhibit decreased migration towards SPHK1-knockdown cells (Figure S2H) compared to the corresponding controls. In line with our previous observations, human monocytes showed an increased chemoattraction when treated with the S1PR2-specific antagonist JTE-013 (Figure S2I). Altogether, our results show that human monocytes have a similar dependency on S1P for their migration as murine macrophages.

Cell motility is stimulated when S1PR1 is coupled to Rac-Cdc42 and inhibited through S1PR2-dependent regulation of Rho protein [6]. Despite the fact that endogenous signaling of S1P/S1PR1 through Rac1/RhoA has been already described, we investigated in a co-culture setting whether S1P secreted by tumor cells is able to signal via Rac1/RhoA in macrophages, thus affecting their chemoattraction properties and inducing their recruitment. Therefore, we pulled down active Rac1 in RAW264.7 cells cultured in media conditioned by LN229 cells for 15 or 30 min. We observed increased Rac1-GTP pull-down when RAW264.7 cells were cultured with LN229-conditioned medium compared to cells cultured in control medium (Figure 2E). To specifically assess the role of S1P on Rac1 activation, we pulled down Rac1-GTP upon either addition of S1P or after treatment with the potent S1PR1 activator CYM-5442 [38]. Our results showed an increased Rac1-GTP pull-down upon treatment with both compounds compared to their corresponding controls (Figure 2F). Conversely, we observed a significant reduction of active Rac1-GTP when RAW264.7 macrophages were cultured in media conditioned by SPHK1-knockdown GB cells compared to the controls (Figure 2G,H). Furthermore, we simultaneously evaluated both Rac1 and RhoA activation upon treatment of RAW264.7 cells with the S1PR2-specific antagonist JTE-013, which enhanced macrophage chemoattraction (Figure 2D). Despite DMSO alone strongly affecting RhoA activation, JTE-013 triggered a significant dose-dependent decrease compared to the solvent control (Figure 2I). DMSO had a minor impact on Rac1 activation, which was significantly increased by the inhibition of S1PR2 with 2 µM JTE-013 (Figure 2I), in line with the higher migration rate of RAW264.7 cells observed before (Figure 2D).

Taken together, these results show that S1P drives macrophage chemoattraction via Rac1/RhoA-mediated signaling.

### 3.3. S1P Shifts TAMs to an Anti-Inflammatory Phenotype, Which Is Rescued by SPHK1 Inhibition

**Figure 3:**
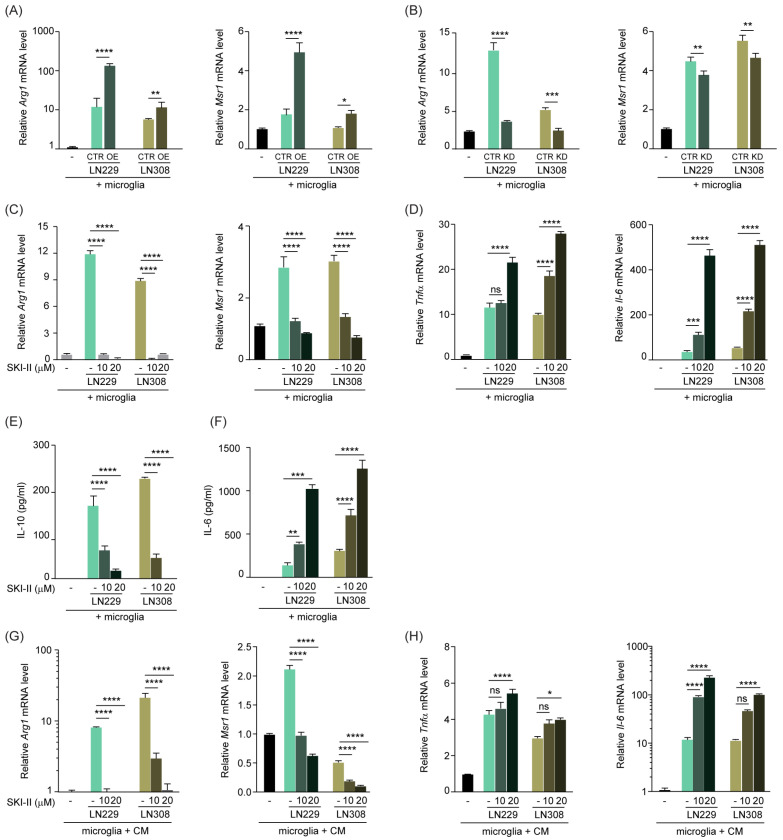
**S1P shifts TAMs to an anti-inflammatory phenotype, which is rescued upon inhibition of SPHK1.** (**A**,**B**) Quantitative RT-PCR analysis of M2 markers Arg1 and Msr1 in murine primary microglia co-cultured with LN229 or LN308 human glioma cells, overexpressing (OE, (**A**)) or knockdown for (KD, (**B**)) SPHK1. (**C**,**D**) Quantitative RT-PCR analysis of Arg1 and Msr1 (**C**) and M1 markers TNFα and Il-6 (**D**) in primary microglia co-cultured with LN229 or LN308, and treated for 24 h with 10 or 20 μM of SPHK1 inhibitor SKI-II or corresponding solvent control. Target genes were normalized to the mean of two housekeeping genes (B2m, Hprt). Data are expressed as fold-change over microglia cells cultured without glioma cells. (**E**,**F**) ELISA of the M2 cytokine IL-10 (**E**) and the M1 cytokine IL-6 (**F**) in cell-free supernatant of murine primary microglia co-cultured with LN229 or LN308, upon treatment with SKI-II for 24 h. Data are expressed as pg/mL as calculated from a standard curve for each cytokine. (**G**,**H**) Quantitative RT-PCR analysis of Arg1 and Msr1 (**G**) and TNFα and Il-6 (**H**) in primary microglia cultured in media (CM) conditioned by LN229 or LN308, and treated for 24 h with 10 or 20 μM of SKI-II or corresponding control. Target genes were normalized to the mean of two housekeeping genes (B2m, Hprt). Data are expressed as fold-change over microglia cells cultured without glioma cells. Mean with SEM are shown, n = 3, ns: not significant, * *p* < 0.05, ** *p* < 0.01, *** *p* < 0.001, **** *p* < 0.0001 by one-way ANOVA.

Microglia and macrophages are remarkably plastic cells that, following an appropriate stimulation, undergo rapid changes and contribute to immune responses. In order to investigate whether, besides affecting the migration properties of microglia and macrophages, S1P also acts as a stimulus to alter their activation status, we set up a co-culture system with murine primary microglia cells and tumor cells either overexpressing or downregulating SPHK1. We analyzed via qPCR the expression level of anti-inflammatory (M2) genes in the microglia and showed that, in these co-culture settings, microglia presented an increased expression of the M2 marker Arg1 compared with cells cultured in the absence of tumor. This effect was further enhanced when microglia were co-cultured with either LN229 or LN308 cells overexpressing SPHK1 (Figure 3A). SPHK1 overexpression in LN229 or LN308 cells also induced another M2 marker, Msr1, in co-cultured microglia (Figure 3A). Conversely, silencing of SPHK1 resulted in a strongly decreased expression of the two M2 markers in microglia, compared to cells cultured with control tumor cells (Figure 3B).

We then adopted the small molecule inhibitor SKI-II, which specifically blocks the binding of sphingosine and ATP to SPHK1 [39] and is therefore widely used as an SPHK1 inhibitor [40]. In line with our previous results, the drug-mediated SPHK1 inhibition resulted in a strong reduction of Arg1 and Msr1 expression (Figure 3C and Figure S3A) in the tumor-microglia co-culture. Interestingly, this reduction was paralleled by a significant increase in the expression of the M1 markers Tnfα and Il-6 (Figure 3D and Figure S3B), in a dose-dependent fashion. Furthermore, we co-cultured microglia with tumor cells and measured the cytokines IL-10 and IL-6, characteristic for M2 and M1, respectively, upon SKI-II administration. With such treatment, we observed a similar trend of IL-10 reduction and IL-6 increase as for the transcript levels (Figure 3E,F and Figure S3C,D). The cytokine levels measured in the conditioned media of the tumor cells were very low compared to the co-cultures and were not affected by the treatment

In order to test whether a potential physical interaction between microglia and tumor cells in the above-mentioned assays was crucial or not for microglia stimulation, we cultured microglia in the conditioned medium of tumor cells that were exposed to the SKI-II drug. Also in these settings, the increased M2 phenotype triggered by the tumor cells was reverted upon SKI-II treatment, as evident by reduced expression of Arg1 and Msr1 and enhanced expression of Tnfα and Il-6 (Figure 3G,H and Figure S3E,F). These results are in line with the hypothesis that soluble factors secreted by the tumor cells indeed drive the phenotypic changes observed in microglia.

Furthermore, we established a co-culture system with RAW264.7 macrophages and genetically modified LN229 or LN308 cells. Using this system, we showed that the expression of the M2 marker Arg1 was significantly increased or decreased upon SPHK1 overexpression or down-regulation, respectively (Figure S3G,H). Moreover, upon SKI-II treatment, we observed a significant downregulation of Arg1 expression in the RAW264.7 cells co-cultured with all the tumor cell lines tested (Figure S3I), accompanied by increased gene expression of the M1 marker Tnfα (Figure S3J) and IL-6 cytokine level in the cell-free supernatant of RAW264.7 cells (Figure S3K).

Ultimately, we adopted a syngeneic culture system where murine primary microglia were co-cultured with TU-9648 [41], a glioma cell line derived from spontaneous mouse brain tumors, in the presence of the SKI-II inhibitor or its solvent control. The analysis of M2 and M1 marker expression in this system robustly confirmed our previous observations (Figure S3L–O). Moreover, we obtained similar results when RAW264.7 macrophages were co-cultured with the murine TU-9648 cell line and treated with SKI-II (Figure S3P,Q).

Taken together, these data support the hypothesis that S1P secreted by the tumor cells not only recruits TAMs but also alters their phenotype and induces a tumor-supporting state, which can be reverted through its down-modulation.

### 3.4. S1P Reduces NFkB-Mediated Inflammation via S1PR1- and S1PR2-Dependent Signaling

**Figure 4:**
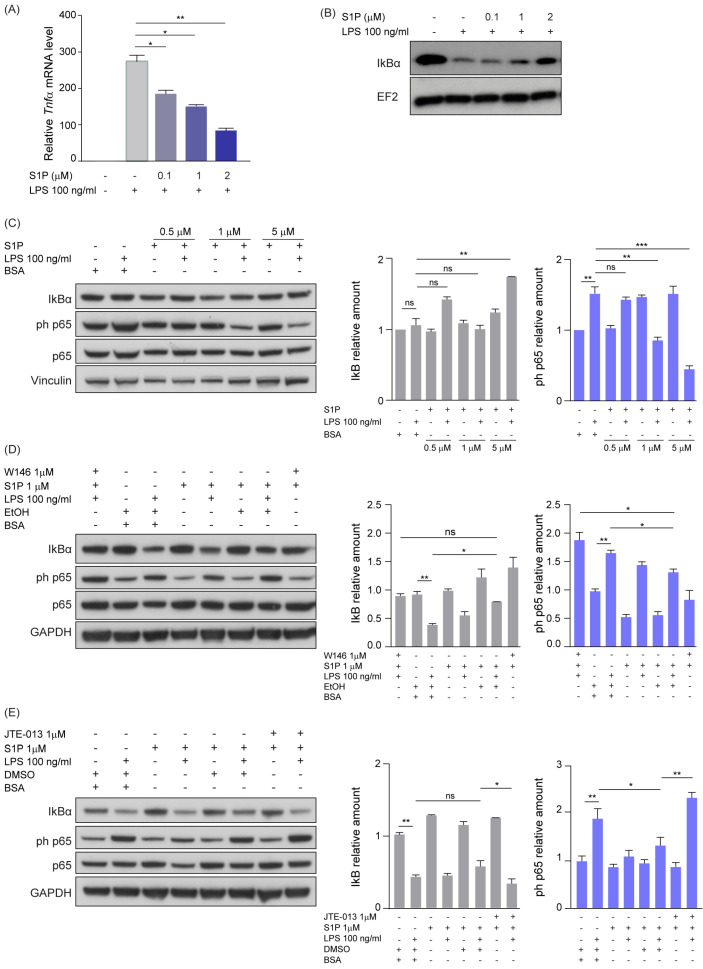
**S1P reduces NFkB-mediated inflammation via S1PR1- and S1PR2-dependent signaling.** (**A**) Quantitative RT-PCR analysis of Tnfα in primary microglia co-treated with LPS and increasing concentration of S1P (0–2 μM) for 5 h. The target gene was normalized to the mean of two housekeeping genes (B2m, Hprt). Data are expressed as fold-change over cells treated with solvent control. (**B**) Western blot analysis of IκBα amounts in murine primary microglia treated with LPS and increasing concentrations of S1P (0–2 μM) for 1 h. EF2 served as loading control. (**C**) Western blot analysis (**left**) and corresponding quantification (**right**) of NFkB activation in RAW264.7 macrophages co-treated with LPS (100 ng/μL) and increasing concentrations of S1P for 1 h. (**D**,**E**) Western blot analysis and corresponding quantification of NFkB activation in RAW264.7 macrophages pre-treated with either S1PR1 (W146, (**D**)) or S1PR2 (JTE013, (**E**)) antagonists and subsequently co-treated with LPS (100 ng/μL) and S1P (1 μM) for 1 h. EtOH or DMSO served as solvent controls. Vinculin and GAPDH served as loading controls. Mean with SEM are shown, n = 3, ns: not significant, * *p* < 0.05, ** *p* < 0.01, *** *p* < 0.001 by Kruskal–Wallis test.

In order to elucidate the molecular mechanism mediating the shift of TAMs towards a pro-inflammatory phenotype and on the basis of previous evidence correlating S1P with NFκB [42,43], we investigated the effect of S1P on the activation of NFkB signaling. We therefore treated either murine primary microglia or RAW264.7 macrophages for 5 h with LPS and increasing concentrations of soluble S1P. Quantitative PCR analysis showed that the presence of S1P efficiently decreased the expression of LPS-induced M1 marker genes Tnfα and iNos both in microglia and RAW264.7 macrophages (Figure 4A and Figure S4A,B). Moreover, we observed a similar effect when analyzing the amount of nitric oxide, a pro-inflammatory metabolite, in RAW264.7 macrophages treated with S1P and LPS (Figure S4C). Furthermore, we analyzed the amount of the NFkB inhibitor alpha (IkBα) in protein lysates of murine primary microglia treated for 1 h with LPS and exposed to increasing concentrations of S1P. Our results showed that S1P was able to reduce LPS-induced IκBα degradation in a dose-dependent manner (Figure 4B). We observed a similar dose-dependent IkBα degradation when treating microglia for 1 h with SEW-2871, a potent and selective S1PR1 agonist [44], in combination with LPS (Figure S4D).

Next, we treated RAW264.7 macrophages for 1 h with LPS and increasing concentrations of S1P and performed immunoblot analyses of IkBα and the NFkB signaling mediator phosphorylated p65 (phospho-p65). As we observed a dose-dependent increase of total IκBα in the samples treated with LPS and S1P (Figure 4C), a role of S1P in limiting LPS-induced IκBɑ degradation was suggested. This effect was paralleled by a progressive decrease of phospho-p65 upon treatment with LPS and increasing levels of S1P (Figure 4C).

In order to clarify through which receptor S1P is exerting its signaling, we inhibited the S1PR1 and S1PR2 receptors by using the W146 and JTE-013 drugs in RAW264.7 cells. Inhibition of S1PR1 rescued the S1P-induced effect, as observed by the increased phospho-p65 and reduced IkBα amounts upon W146 treatment (Figure 4D). S1PR2 inhibition by JTE-013 treatment decreased IκBα and increased phospho-p65 levels (Figure 4E). Taken together, these results suggest that S1P inhibits the pro-inflammatory phenotype of macrophages/microglia via both S1PR1 and S1PR2.

### 3.5. S1P Exerts a Supporting Role in Orthotopic Brain Tumors and Correlates with Worse Outcome in GB Patients

**Figure 5:**
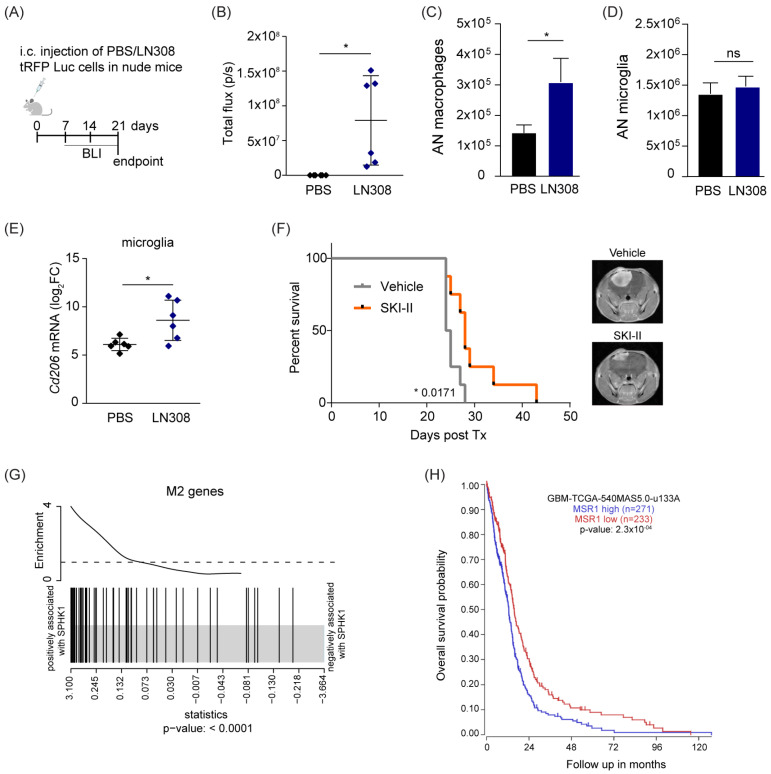
**S1P is pro-tumorigenic in vivo and in human GB.** (**A**) Schematic representation of the in vivo study. (**B**) Bioluminescent measurement of tumor growth at the endpoint. Intensity is expressed as total flux (photons per second). (**C**,**D**) Absolute counts (AN) of macrophages (CD11b+/CD45high/CD49dhigh, (**C**)) and microglia (CD11b+/CD45low/CD49dlow, (**D**)) as determined by flow cytometry. (**E**) Quantitative RT-PCR analysis of the M2 marker Cd206 in FACS-sorted microglia. The target gene was normalized to the mean of two housekeeping genes (B2m, Hprt). Data are expressed as log2FC normalized on PBS mice control. Mean with SD are shown, n = 6, ns: not significant * *p* < 0.05 by unpaired *t*-test. (**F**) Kaplan–Meier survival curves of mice orthotopically implanted with LN229 cells and treated with SKI-II inhibitor or vehicle (n = 8). Representative MRI images of mouse brains are shown. (**G**) Gene set enrichment analysis (GSEA) of the M2 gene signature as reported in Engler et al., 2012 [46] on TCGA Affimetrix data of a GB dataset. The barcode plot shows a positive association between mRNA expression of SPHK1 and the M2 genes. (**H**) Kaplan–Meier plot estimates overall survival in patients with glioblastoma divided into high and low MSR1 expression. Data analyzed using the Affymetrix 540 MASS 5.0-u133 array available in the online R2: Genomics Analysis and Visualization Platform (http://r2.amc.nl, accessed on 10 October 2022). *p* value in Kaplan–Meier results from scan modus test.

Our in vitro studies showed that S1P impacts microglia and macrophages recruitment and polarization. To verify the relevance of these findings in vivo, we intracranially injected tRFP-labeled LN308 cells in nude mice (Figure 5A). Bioluminescent measurements performed at different time points after injection confirmed orthotopic tumor growth in the LN308-injected mice (Figure 5B). To quantify infiltration of TAMs into these brain tumors, we analyzed by flow cytometry on dissociated brain cells (Figure S5A) specific surface markers that discriminate resident microglia from infiltrating macrophages [45]. This analysis showed an increased number of bone marrow-derived macrophages (BMDM, CD11b+/CD45high/CD49dhigh) in the brain of tumor-injected mice compared to the control group (Figure S5B), whereas the total number of microglia (CD11b+/CD45low/CD49dlow) was not altered (Figure 5C,D). To further investigate the phenotypic features of TAMs, we FACS-sorted the resident microglia and analyzed the expression of Tnfα and Cd206 as markers of M1 and M2 phenotype, respectively. Interestingly, we observed a significant increase in the expression of Cd206 in LN308-injected mice compared to controls (Figure 5E), whereas no significant difference was detected in the level of the pro-inflammatory marker Tnfα (Figure S5C). Altogether, these in vivo results mirror our previous in vitro data, thus demonstrating a tumor-mediated recruitment of macrophages to the tumor site and the induction of an anti-inflammatory phenotype in the TAM compartment.

To more specifically assess the role of S1P in tumor growth, we orthotopically injected LN229 cells in nude mice and compared their overall survival in the presence of the S1P inhibitor SKI-II or the corresponding vehicle (Figure 5F). We observed a significant survival benefit from SKI-II administration compared with control mice, indicating that S1P supports tumor growth, and its inhibition can be an effective strategy in treatment of GB. We then interrogated whether these observations can be extended to human tumors. To address this, we exploited the TCGA GB data previously explored (Figure 2) and performed GSEA to investigate the M2 gene signature previously defined by Engler et al. [46]. Interestingly, we observed that this M2 signature had a positive association with SPHK1 expression (Figure 5G). Moreover, higher expression of the M2 marker MSR1 was associated with worse outcome in the GB cohort (Figure 5H). Conversely, a higher level of the M1 marker NOS1 led to a better prognosis (Figure S5D). Based on these results, it became clear that the relationship between increased tumor aggressiveness and a higher anti-inflammatory phenotype of TAMs can be explained by the expression levels of the SPHK1 gene. Indeed, the analysis of SPHK1 expression across the main four GB subtypes showed that the mesenchymal (ME) subtype, characterized by shorter survival rates and higher infiltration of non-neoplastic cells [47], had the highest SPHK1 levels and was associated with an enriched M2 gene signature (Figure S5E,F).

Taken together, our data show that S1P plays a crucial role in the GB microenvironment, with a particular focus on the macrophage/microglia compartment. We also show that S1P signals through S1PR1 and S1PR2 receptors, and that their pharmacological inhibition counteracts the effect triggered by the lipid. Our results therefore propose a new perspective to obtain more effective GB treatment options and open the way for the development of specific novel therapeutic targets.

## 4. Discussion

GB is the most common malignant brain tumor among adults, characterized by rapid tumor recurrence and a poor prognosis [48]. Signaling pathways involved in the rapid tumor recurrence could contribute to the improvement of current therapeutic intervention techniques and therefore need to be investigated. The bioactive metabolite S1P is present at high concentrations in the brain [49] and promotes invasiveness of GB cells through its receptors [18,50]. The balance between S1P levels and its precursor ceramide defines the so-called “sphingolipid rheostat”, based on which cells are directed towards either apoptosis or survival. SPHK 1 and 2, the enzymes deputed to the phosphorylation of sphingosine into S1P, are key regulators of this rheostat [51]. Targeting the SPHK/S1P/S1PR signaling axis as a therapeutic strategy against cancer has been intensively investigated by a number of groups [51,52]. The majority of therapeutic strategies have so far focused on addressing cancer cell proliferation and invasion, but a great amount of evidence suggests that microenvironment plays a crucial role in the development and progression of the tumor [53,54]. Therefore, new strategies targeting microenvironmental components, such as microglia, macrophages, astrocytes, and endothelial cells, are strongly needed.

In this study, we focused on the effects of S1P on regulating the cross-talk between tumor cells and TAMs, providing the notion that inhibiting S1P secretion and simultaneously blocking S1P-mediated signaling in TAMs strongly affects tumor development. Our observation that higher SPHK1 expression results in worse prognosis is in accordance with previous findings showing that increased SPHK1, but not SPHK2, correlates with lower progression-free survival in GB and has been associated with increased glioma grade [55,56]. SPHK1 is upregulated in multiple types of cancer including melanoma [57], papillary thyroid carcinoma [58], non-small cell lung cancer [59], triple-negative breast cancer [60], and colorectal cancer [61]. Moreover, we found a positive association between SPHK1 and microglial gene expression and showed in vitro the fundamental role of S1P as a promoter of macrophage chemoattraction in co-culture systems. Our findings are in line with previous evidence demonstrating that extracellular S1P is a potent chemoattractant for many different types of normal and malignant cells. Interestingly, S1P released from apoptotic leukemic cells attracts monocytes as efficiently as the monocyte chemoattractant protein-1 (MCP-1/CCL2) [62]. Chemoattractive properties of S1P are confirmed in several other models including gastric cancer [63], leukemia [64], lung cancer [65], glioblastoma [66], ovarian cancer [67], rhabdomyosarcoma [68], breast cancer [69], and acute T-cell leukemia [70]. In line with previous findings describing an opposite effect of S1PR1 and S1PR2 on cell migration [36,71], we showed a complementary function of these two receptors in recruiting macrophages. Moreover, our results linked the S1PR1-coupled Gi protein with an activation of GTP-Rac1 and S1P-induced macrophage migration. On the other hand, we showed that S1PR2, which is known to couple with Gi, Gq, and G12/13 pathways [72], activates RhoA and inhibits S1P-induced macrophage migration. These results strongly indicate that integration of counteracting signals from the Gi-Rac1 and the G12/13-RhoA pathways works in a coordinated manner and regulates cell migration upon activation of S1PR1 and S1PR2, respectively. As S1P signaling through its receptors on macrophages seems to be a potent driving factor for recruiting macrophages in GB, S1PR1/2 expression in microglial cells and S1P secretion by tumor cells likely contribute to tumor aggressiveness.

We observed that modulating SPHK1 significantly affected the TAMs phenotype, with a substantial shift towards a pro-inflammatory status upon SPHK1 inhibition. Interestingly, we proved that the activity of S1P does not require a physical interaction between tumor and the microenvironment. These results are in line with previous pieces of evidence showing that, in several models, including breast cancer, S1P was found to induce an anti-inflammatory phenotype in macrophages [73]. Furthermore, a better response to anti-PD1 and other immune checkpoint inhibitors as well as reduced tumor growth has been observed in murine models of melanoma, breast, and colon cancer upon SPHK1 silencing [57].

Our results also showed that S1P is involved in the regulation of inflammation via suppression of NFκB. Indeed, S1P secreted by tumor cells inhibited LPS-mediated M1 phenotype of macrophage/microglia, as observed by decreased expression of LPS-induced M1 markers such as Tnfα and iNos and reduced nitric oxide production. This modulation is mediated by inhibition of the NFkB pathway, as indicated by the reduced IκBα degradation and increased p65 phosphorylation. This is in line with several studies correlating S1P with NFκB, p38 MAPK, and JNK signaling pathways [42,43,69]. It was already known that intracellular S1P can activate NFκB; recent studies indicate that an extracellular pool of S1P can also mediate this activation mainly through S1PR1-3 [74]. Interestingly, previous results suggest S1P as a signaling molecule and/or as a cofactor of TRAF2 E3 ubiquitin ligase, acting as a link between TNF signaling and NFκB activation [75]. In melanoma cells, extracellular S1P activated NFκB, and this was correlated with expression of actin-binding protein FlnA [74], an interaction partner of SPHK1 [76] and TRAF2 [77]. In inflammation-associated colon cancer, S1P was crucial for the production of the NFκB-dependent cytokine IL-6, essential for STAT3 activation [78]. The connection between S1PR1 and STAT3 activation was fundamental in lymphoma, adenocarcinoma, melanoma, breast, and prostate cancers [78], and targeting S1PR1 to decrease expression of STAT3-regulated genes resulted in inhibition of tumor progression [79].

In agreement with the notion that TAMs are attracted to tumor cells and help create an immunosuppressive milieu that favors glioma growth [80], we showed an increased recruitment of BMDM in mice transplanted with astrocytoma cell lines, and an increased anti-inflammatory phenotype in resident microglia, which also correlated with shorter survival, in accordance with previous findings [81]. These results are in agreement with the current notion that M2-polarized microglia in glioma exhibit reduced phagocytic activity and secretion of immunosuppressive cytokines and are less capable of stimulating T-cell proliferation ex vivo [82]. Similar findings were obtained in glioma mouse models [83] and glioma patients [84].

Our observations, showing that a more pronounced anti-inflammatory phenotype correlates with higher SPHK1 expression in human TCGA data, ultimately reinforce the hypothesis that S1P may represent one of the glioma-derived factors able to “educate” TAMs to create a favorable microenvironment for glioma growth. Interestingly, we provided evidence that the aggressive mesenchymal (MES) subtype of GB showed the highest SPHK1 expression. This is in accordance with previous findings, describing that MES glioma cell differentiation status correlates with an enrichment of macrophages/microglia [47,85] and with more M2 TAMs. Specifically, expression of M2 markers CD163 and CD204 by macrophages/microglia infiltrating glioma was significantly higher in grade IV glioma compared to World Health Organization (WHO) grades II and III, indicating that M2 polarization of glioma-infiltrating microglia correlates with a more malignant histologic grade [86].

Our in vivo data strongly suggest that targeting the SPHK1/S1P/S1PR axis might be a valuable approach in counteracting tumor development. Targeting S1PRs on the TAM compartment could represent an important therapeutic intervention. Fingolimod (FTY720), a sphingosine analog driving the internalization of S1PR1, decreased the recruitment of macrophages to the brain tumor microenvironment and induced a pro-inflammatory phenotype [87]. This downregulation of S1PR1 and the consequent retention of lymphocytes within the lymph nodes has been exploited for the treatment of the relapsing and remitting form of multiple sclerosis, in order to alleviate autoimmunity symptoms [88]. This aspect might be beneficial for high grade glioma patients if the drug is administered prior to radio- and chemotherapy in order to prevent lymphocytes from circulation, thus avoiding the immunosuppressive effects of radiation (NCT02490930) [89].

## 5. Conclusions

Taken together, our results provide new evidence elucidating the link between glioma and infiltrating TAMs, pointing towards a key role played by S1P in regulating this complex crosstalk, and thus providing an imperative for adopting S1P blocking agents as factors inhibiting the pro-tumorigenic role of TAMs.

## Data Availability

The data that support the findings of this study are available in the online R2: Genomics Analysis and Visualization Platform (http://r2.amc.nl, accessed on 10 October 2022) and in the following resources: Butovsky et al., 2014 [30] and Engler et al., 2012 [46].

## Supplementary Materials

The following supporting information can be downloaded at: https://www.mdpi.com/article/10.3390/cancers15020479/s1, Figure S1: High SPHK1 expression strongly correlates with higher microglia recruitment; Figure S2: SPHK1 modulation is effective both at the expression and protein level and results in altered S1P secretion and higher monocytes chemoattraction; Figure S3: S1P shifts TAMs to an anti-inflammatory phenotype, whereas inhibition of SPHK1 activity reverts this phenotype into a pro-inflammatory one; Figure S4: S1P reduces LPS-induced inflammation; Figure S5: Increased expression of pro-inflammatory genes correlates with a better survival in GB; Figure S6: Original WB files; Table S1: Cell lines; Table S2: Plasmids; Table S3: Short hairpin RNA; Table S4: Primers; Table S5: Antibodies; Table S6: Measurements of S1P derivatives analyzed by UPLC-ESI-(QqQ)-MS; Table S7: Multiple reaction monitoring (MRM) for S1P measurements.

## References

[1] Louis D.N., Perry A., Reifenberger G., Von Deimling A., Figarella-Branger D., Cavenee W.K., Ohgaki H., Wiestler O.D., Kleihues P., Ellison D.W. (2016). The 2016 World Health Organization Classification of Tumors of the Central Nervous System: A summary. Acta Neuropathol..

[2] Quail D.F., Joyce J.A. (2013). Microenvironmental regulation of tumor progression and metastasis. Nat. Med..

[3] Buonfiglioli A., Hambardzumyan D. (2021). Macrophages and microglia: The cerberus of glioblastoma. Acta Neuropathol. Commun..

[4] Mildenberger W., Stifter S.A., Greter M. (2022). Diversity and function of brain-associated macrophages. Curr. Opin. Immunol..

[5] Hume D.A. (2015). The many alternative faces of macrophage activation. Front. Immunol..

[6] Pyne N.J., Pyne S. (2010). Sphingosine 1-phosphate and cancer. Nat. Rev. Cancer.

[7] Pyne S., Pyne N.J. (2011). Translational aspects of sphingosine 1-phosphate biology. Trends Mol. Med..

[8] Blaho V.A., Hla T. (2014). An update on the biology of sphingosine 1-phosphate receptors. J. Lipid Res..

[9] Pyne S., Adams D.R., Pyne N.J. (2016). Sphingosine 1-phosphate and sphingosine kinases in health and disease: Recent advances. Prog. Lipid Res..

[10] Singh S.K., Spiegel S. (2020). Sphingosine-1-phosphate signaling: A novel target for simultaneous adjuvant treatment of triple negative breast cancer and chemotherapy-induced neuropathic pain. Adv. Biol. Regul..

[11] Hernandez-Coronado C.G., Guzman A., Castillo-Juarez H., Zamora-Gutierrez D., Rosales-Torres A.M. (2019). Sphingosine-1-phosphate (S1P) in ovarian physiology and disease. Ann. Endocrinol..

[12] Sukocheva O.A., Furuya H., Ng M.L., Friedemann M., Menschikowski M., Tarasov V.V., Chubarev V.N., Klochkov S.G., Neganova M.E., Mangoni A.A. (2020). Sphingosine kinase and sphingosine-1-phosphate receptor signaling pathway in inflammatory gastrointestinal disease and cancers: A novel therapeutic target. Pharmacol. Therapeut..

[13] Maceyka M., Rohrbach T., Milstien S., Spiegel S. (2020). Role of Sphingosine Kinase 1 and Sphingosine-1-Phosphate Axis in Hepatocellular Carcinoma. Handb. Exp. Pharmacol..

[14] Tea M.N., Poonnoose S.I., Pitson S.M. (2020). Targeting the Sphingolipid System as a Therapeutic Direction for Glioblastoma. Cancers.

[15] Chongsathidkiet P., Jackson C., Koyama S., Loebel F., Cui X., Farber S.H., Woroniecka K., Elsamadicy A.A., Dechant C.A., Kemeny H.R. (2018). Sequestration of T cells in bone marrow in the setting of glioblastoma and other intracranial tumors. Nat. Med..

[16] Sharma R.K. (2019). Understanding the Role of Sphingosine 1-Phosphate in Regulating the Microglia and Glioma Interactions. Master’s Thesis.

[17] Osada M., Yatomi Y., Ohmori T., Ikeda H., Ozaki Y. (2002). Enhancement of sphingosine 1-phosphate-induced migration of vascular endothelial cells and smooth muscle cells by an EDG-5 antagonist. Biochem. Biophys. Res. Commun..

[18] Bien-Möller S., Lange S., Holm T., Böhm A., Paland H., Küpper J., Herzog S., Weitmann K., Havemann C., Vogelgesang S. (2016). Expression of S1P metabolizing enzymes and receptors correlate with survival time and regulate cell migration in glioblastoma multiforme. Oncotarget.

[19] Sadik A., Patterson L.F.S., Öztürk S., Mohapatra S.R., Panitz V., Secker P.F., Pfänder P., Loth S., Salem H., Prentzell M.T. (2020). IL4I1 Is a Metabolic Immune Checkpoint that Activates the AHR and Promotes Tumor Progression. Cell.

[20] Yang Y., Torta F., Arai K., Wenk M.R., Herr D.R., Wong P.T., Lai M.K. (2016). Sphingosine kinase inhibition ameliorates chronic hypoperfusion-induced white matter lesions. Neurochem. Int..

[21] Saura J., Tusell J.M., Serratosa J. (2003). High-yield isolation of murine microglia by mild trypsinization. Glia.

[22] Wirthschaft P., Bode J., Soni H., Dietrich F., Krüwel T., Fischer B., Knobbe-Thomsen C.B., Rossetti G., Hentschel A., Mack N. (2019). RhoA regulates translation of the Nogo-A decoy SPARC in white matter-invading glioblastomas. Acta Neuropathol..

[23] Ritchie M.E., Belinda P., Wu D., Hu Y., Law C.W., Shi W., Smyth G.K. (2015). limma powers differential expression analyses for RNA-sequencing and microarray studies. Nucleic Acids Res..

[24] Wu D., Smyth G.K. (2012). Camera: A competitive gene set test accounting for inter-gene correlation. Nucleic Acids Res..

[25] Robinson M.D., McCarthy D.J., Smyth G.K. (2010). EdgeR: A Bioconductor package for differential expression analysis of digital gene expression data. Bioinformatics.

[26] Hothorn T., Lausen B. (2003). On the exact distribution of maximally selected rank statistics. Comput. Stat. Data Anal..

[27] Hothorn T., Zeileis A. (2008). Generalized Maximally Selected Statistics. Biometrics.

[28] Lausen B., Schumacher M. (1992). Maximally Selected Rank Statistics. Biometrics.

[29] Guillermet-Guibert J., Davenne L., Pchejetski D., Saint-Laurent N., Brizuela L., Guilbeau-Frugier C., Delisle M.B., Cuvillier O., Susini C., Bousquet C. (2009). Targeting the sphingolipid metabolism to defeat pancreatic cancer cell resistance to the chemotherapeutic gemcitabine drug. Mol. Cancer Ther..

[30] Butovsky O., Jedrychowski M.P., Moore C.S., Cialic R., Lanser A.J., Gabriely G., Koeglsperger T., Dake B., Wu P.M., Doykan C.E. (2014). Identification of a unique TGF-beta-dependent molecular and functional signature in microglia. Nat. Neurosci..

[31] Elmore M.R.P., Najafi A.R., Koike M.A., Dagher N.N., Spangenberg E.E., Rice R.A., Kitazawa M., Matusow B., Nguyen H., West B.L. (2014). Colony-Stimulating Factor 1 Receptor Signaling Is Necessary for Microglia Viability, Unmasking a Microglia Progenitor Cell in the Adult Brain. Neuron.

[32] Payne J., Maher F., Simpson I., Mattice L., Davies P. (1997). Glucose transporter Glut 5 expression in microglial cells. Glia.

[33] Bennett M.L., Bennett F.C., Liddelow S.A., Ajami B., Zamanian J.L., Fernhoff N.B., Mulinyawe S.B., Bohlen C.J., Adil A., Tucker A. (2016). New tools for studying microglia in the mouse and human CNS. Proc. Natl. Acad. Sci. USA.

[34] Jones B.A., Beamer M., Ahmed S. (2010). Fractalkine/CX3CL1: A Potential New Target for Inflammatory Diseases. Mol. Interv..

[35] Haynes S.E., Hollopeter G., Yang G., Kurpius D., Dailey M.E., Gan W.-B., Julius D. (2006). The P2Y12 receptor regulates microglial activation by extracellular nucleotides. Nat. Neurosci..

[36] Bryan A.M., Del Poeta M. (2018). Sphingosine-1-phosphate receptors and innate immunity. Cell Microbiol..

[37] Liu J., Zhao J., Lee J.F., Gartung A., Jawadi H., Zhang W., Lominadze D., Lee M.-J. (2013). 3-amino-4-(3-hexylphenylamino)-4-oxobutyl phosphonic acid (W146), a Selective Antagonist of Sphingosine-1-phospahte Receptor Subtype 1, Enhances AMD3100-stimulated Mobilization of Hematopoietic Stem Progenitor Cells in Animals. J. Biochem. Pharmacol. Res..

[38] Gonzalez-Cabrera P.J., Jo E., Sanna M.G., Brown S., Leaf N., Marsolais D., Schaeffer M.-T., Chapman J., Cameron M., Guerrero M. (2008). Full pharmacological efficacy of a novel S1P1 agonist that does not require S1P-like headgroup interactions. Mol. Pharmacol..

[39] French K.J., Schrecengost R.S., Lee B.D., Zhuang Y., Smith S.N., Eberly J.L., Yun J., Smith C.D. (2003). Discovery and evaluation of inhibitors of human sphingosine kinase. Cancer Res..

[40] Xu C.-Y., Liu S.-Q., Qin M.-B., Zhuge C.-F., Qin L., Qin N., Lai M.-Y., Huang J.-A. (2017). SphK1 modulates cell migration and EMT-related marker expression by regulating the expression of p-FAK in colorectal cancer cells. Int. J. Mol. Med..

[41] Weissenberger J., Priester M., Bernreuther C., Rakel S., Glatzel M., Seifert V., Kögel D. (2010). Dietary Curcumin Attenuates Glioma Growth in a Syngeneic Mouse Model by Inhibition of the JAK1,2/STAT3 Signaling Pathway. Clin. Cancer Res..

[42] Park S.J., Lee K.P., Kang S., Lee J., Sato K., Chung H.Y., Okajima F., Im D.-S. (2014). Sphingosine 1-phosphate induced anti-atherogenic and atheroprotective M2 macrophage polarization through IL-4. Cell. Signal..

[43] Hughes J.E., Srinivasan S., Lynch K.R., Proia R.L., Ferdek P., Hedrick C.C. (2008). Sphingosine-1-phosphate induces an antiinflammatory phenotype in macrophages. Circ. Res..

[44] Hale J.J., Lynch C.L., Neway W., Mills S.G., Hajdu R., Keohane C.A., Rosenbach M.J., Milligan J.A., Shei G.-J., Parent S.A. (2004). A rational utilization of high-throughput screening affords selective, orally bioavailable 1-benzyl-3-carboxyazetidine sphingosine-1-phosphate-1 receptor agonists. J. Med. Chem..

[45] Bowman R.L., Klemm F., Akkari L., Pyonteck S.M., Sevenich L., Quail D.F., Dhara S., Simpson K., Gardner E.E., Iacobuzio-Donahue C.A. (2016). Macrophage Ontogeny Underlies Differences in Tumor-Specific Education in Brain Malignancies. Cell Rep..

[46] Engler J.R., Robinson A.E., Smirnov I., Hodgson J.G., Berger M.S., Gupta N., James C.D., Molinaro A., Phillips J.J. (2012). Increased microglia/macrophage gene expression in a subset of adult and pediatric astrocytomas. PLoS ONE.

[47] Wang Q.H., Hu B.L., Hu X., Kim H., Squatrito M., Scarpace L., deCarvalho A.C., Lyu S., Li P., Li Y. (2017). Tumor Evolution of Glioma-Intrinsic Gene Expression Subtypes Associates with Immunological Changes in the Microenvironment. Cancer Cell.

[48] Anton K., Baehring J.M., Mayer T. (2012). Glioblastoma multiforme: Overview of current treatment and future perspectives. Hematol./Oncol. Clin. North Am..

[49] Paugh B.S., Bryan L., Paugh S., Wilczynska K.M., Alvarez S.M., Singh S.K., Kapitonov D., Rokita H., Wright S., Griswold-Prenner I. (2009). Interleukin-1 regulates the expression of sphingosine kinase 1 in glioblastoma cells. J. Biol. Chem..

[50] Young N., Pearl D.K., Van Brocklynl J.R. (2009). Sphingosine-1-Phosphate Regulates Glioblastoma Cell Invasiveness through the Urokinase Plasminogen Activator System and CCN1/Cyr61. Mol. Cancer Res..

[51] Shida D., Takabe K., Kapitonov D., Milstien S., Spiegel S. (2008). Targeting SphK1 as a new strategy against cancer. Curr. Drug Targets.

[52] Murph M., Mills G.B. (2007). Targeting the lipids LPA and S1P and their signalling pathways to inhibit tumour progression. Expert Rev. Mol. Med..

[53] Charles N.A., Holland E.C., Gilbertson R., Glass R., Kettenmann H. (2011). The brain tumor microenvironment. Glia.

[54] Quail D.F., Joyce J.A. (2017). The Microenvironmental Landscape of Brain Tumors. Cancer Cell.

[55] Van Brocklyn J.R., Jackson C.A., Pearl D.K., Kotur M.S., Snyder P.J., Prior T.W. (2005). Sphingosine kinase-1 expression correlates with poor survival of patients with glioblastoma multiforme: Roles of sphingosine kinase isoforms in growth of glioblastoma cell lines. J. Neuropath. Exp. Neur..

[56] Abuhusain H.J., Matin A., Qiao Q., Shen H., Kain N., Day B.W., Stringer B., Daniels B., Laaksonen M.A., Teo C. (2013). A Metabolic Shift Favoring Sphingosine 1-Phosphate at the Expense of Ceramide Controls Glioblastoma Angiogenesis. J. Biol. Chem..

[57] Imbert C., Montfort A., Fraisse M., Marcheteau E., Gilhodes J., Martin E., Bertrand F., Marcellin M., Burlet-Schiltz O., Peredo A.G. (2020). Resistance of melanoma to immune checkpoint inhibitors is overcome by targeting the sphingosine kinase-1. Nat. Commun..

[58] Li J., Zhang B., Bai Y., Liu Y.H., Zhang B.Y., Jin J. (2019). Upregulation of sphingosine kinase 1 is associated with recurrence and poor prognosis in papillary thyroid carcinoma. Oncol. Lett..

[59] Gachechiladze M., Tichy T., Kolek V., Grygarkova I., Klein J., Mgebrishvili G., Kharaishvili G., Janíková M., Smičková P., Cierna L. (2019). Sphingosine kinase-1 predicts overall survival outcomes in non-small cell lung cancer patients treated with carboplatin and navelbine. Oncol. Lett..

[60] Acharya S., Yao J., Li P., Zhang C.Y., Lowery F.J., Zhang Q.L., Guo H., Qu J., Yang F., Wistuba. I.I. (2019). Sphingosine Kinase 1 Signaling Promotes Metastasis of Triple-Negative Breast Cancer. Cancer Res..

[61] Bae G.E., Do S.I., Kim K., Park J.H., Cho S., Kim H.S. (2019). Increased Sphingosine Kinase 1 Expression Predicts Distant Metastasis and Poor Outcome in Patients with Colorectal Cancer. Anticancer Res..

[62] Gude D.R., Alvarez S.E., Paugh S.W., Mitra P., Yu J.D., Griffiths R., Barbour S.E., Milstien S., Spiegel S. (2008). Apoptosis induces expression of sphingosine kinase 1 to release sphingosine-1-phosphate as a “come-and-get-me” signal. Faseb J..

[63] Chumanevich A., Wedman P., Oskeritzian C.A. (2016). Sphingosine-1-Phosphate/Sphingosine-1-Phosphate Receptor 2 Axis Can Promote Mouse and Human Primary Mast Cell Angiogenic Potential through Upregulation of Vascular Endothelial Growth Factor-A and Matrix Metalloproteinase-2. Mediat. Inflamm..

[64] Abdelbaset-Ismail A., Cymer M., Borkowska-Rzeszotek S., Brzeźniakiewicz-Janus K., Rameshwar P., Kakar S.S., Ratajczak J., Ratajczak M.Z. (2019). Bioactive Phospholipids Enhance Migration and Adhesion of Human Leukemic Cells by Inhibiting Heme Oxygenase 1 (HO-1) and Inducible Nitric Oxygenase Synthase (iNOS) in a p38 MAPK-Dependent Manner. Stem Cell Rev. Rep..

[65] Schneider G., Sellers Z.P., Bujko K., Kakar S.S., Kucia M., Ratajczak M.Z. (2017). Novel pleiotropic effects of bioactive phospholipids in human lung cancer metastasis. Oncotarget.

[66] Malchinkhuu E., Sato K., Maehama T., Mogi C., Tomura H., Ishiuchi S., Yoshimoto Y., Kurose H., Okajima F. (2008). S1P(2) receptors mediate inhibition of glioma cell migration through Rho signaling pathways independent of PTEN. Biochem. Biophys. Res. Commun..

[67] Park K.S., Kim M.-K., Lee H.Y., Kim S.D., Lee S.Y., Kim J.M., Ryu S.H., Bae Y.-S. (2007). S1P stimulates chemotactic migration and invasion in OVCAR3 ovarian cancer cells. Biochem. Biophys. Res. Commun..

[68] Schneider G., Bryndza E., Abdel-Latif A., Ratajczak J., Maj M., Tarnowski M., Klyachkin Y.M., Houghton P., Morris A.J., Vater A. (2013). Bioactive Lipids S1P and C1P Are Prometastatic Factors in Human Rhabdomyosarcoma, and Their Tissue Levels Increase in Response to Radio/Chemotherapy. Mol. Cancer Res..

[69] Weigert A., Tzieply N., Von Knethen A., Johann A.M., Schmidt H., Geisslinger G., Brüne B. (2007). Tumor cell apoptosis polarizes macrophages*-*Role of sphingosine-1-phosphate. Mol. Biol. Cell.

[70] Weigert A., Johann A.M., von Knethen A., Schmidt H., Geisslinger G., Brune B. (2006). Apoptotic cells promote macrophage survival by releasing the antiapoptotic mediator sphingosine-1-phosphate. Blood.

[71] Green J.A., Suzuki K., Cho B., Willison L.D., Palmer D., Allen C.D.C., Schmidt T.H., Xu Y., Proia R., Coughlin S.R. (2011). The sphingosine 1-phosphate receptor S1P(2) maintains the homeostasis of germinal center B cells and promotes niche confinement. Nat. Immunol..

[72] Sugimoto N., Takuwa N., Okamoto H., Sakurada S., Takuwa Y. (2003). Inhibitory and stimulatory regulation of Rac and cell motility by the G12/13-Rho and Gi pathways integrated downstream of a single G protein-coupled sphingosine-1-phosphate receptor isoform. Mol. Cell Biol..

[73] Brecht K., Weigert A., Hu J., Popp R., Fisslthaler B., Korff T., Fleming I., Geisslinger G., Brüne B. (2011). Macrophages programmed by apoptotic cells promote angiogenesis via prostaglandin E_2_. FASEB J..

[74] Campos L.S., Rodriguez Y.I., Leopoldino A.M., Hait N.C., Lopez Bergami P., Castro M.G., Sanchez E.S., Maceyka M., Spiegel S., Alvarez S.E. (2016). Filamin A Expression Negatively Regulates Sphingosine-1-Phosphate-Induced NF-kappaB Activation in Melanoma Cells by Inhibition of Akt Signaling. Mol. Cell Biol..

[75] Alvarez S.E., Harikumar K.B., Hait N.C., Allegood J., Strub G.M., Kim E.Y., Maceyka M., Jiang H., Luo C., Kordula T. (2010). Sphingosine-1-phosphate is a missing cofactor for the E3 ubiquitin ligase TRAF2. Nature.

[76] Maceyka M., Alvarez S.E., Milstien S., Spiegel S. (2008). Filamin a links sphingosine kinase 1 and sphingosine-1-phosphate receptor 1 at lamellipodia to orchestrate cell migration. Mol. Cell Biol..

[77] Leonardi A., Ellinger-Ziegelbauer H., Franzoso G., Brown K., Siebenlist U. (2000). Physical and functional interaction of filamin (actin-binding protein-280) and tumor necrosis factor receptor-associated factor 2. J. Biol. Chem..

[78] Lee H., Deng J., Kujawski M., Yang C., Liu Y., Herrmann A., Kortylewski M., Horne D., Somlo G., Forman S. (2010). STAT3-induced S1PR1 expression is crucial for persistent STAT3 activation in tumors. Nat. Med..

[79] Liu Y., Deng J.H., Wang L., Lee H., Armstrong B., Scuto A., Kowolik C., Weiss L.M., Forman S., Yu H. (2012). S1PR1 is an effective target to block STAT3 signaling in activated B cell-like diffuse large B-cell lymphoma. Blood.

[80] Chia K., Mazzolini J., Mione M., Sieger D. (2018). Tumor initiating cells induce Cxcr4-mediated infiltration of pro-tumoral macrophages into the brain. Elife.

[81] Tian Y.X., Ke Y.Q., Ma Y.X. (2020). High expression of stromal signatures correlated with macrophage infiltration, angiogenesis and poor prognosis in glioma microenvironment. Peerj.

[82] Hussain S.F., Yang D., Suki D., Aldape K., Grimm E., Heimberger A.B. (2006). The role of human glioma-infiltrating microglia/macrophages in mediating antitumor immune responses. Neuro-Oncology.

[83] Gabrusiewicz K., Ellert-Miklaszewska A., Lipko M., Sielska M., Frankowska M., Kaminska B. (2011). Characteristics of the Alternative Phenotype of Microglia/Macrophages and its Modulation in Experimental Gliomas. PLoS ONE.

[84] Rodrigues J.C., Gonzalez G.C., Zhang L., Ibrahim G., Kelly J.J., Gustafson M.P., Lin Y., Dietz A.B., Forsyth P.A., Yong V.W. (2010). Normal human monocytes exposed to glioma cells acquire myeloid-derived suppressor cell-like properties. Neuro-Oncology.

[85] Bhat K.P.L., Balasubramaniyan V., Vaillant B., Ezhilarasan R., Hummelink K., Hollingsworth F., Wani K., Heathcock L., James J.D., Goodman L.D. (2013). Mesenchymal differentiation mediated by NF-kappaB promotes radiation resistance in glioblastoma. Cancer Cell.

[86] Komohara Y., Ohnishi K., Kuratsu J., Takeya M. (2008). Possible involvement of the M2 anti-inflammatory macrophage phenotype in growth of human gliomas. J. Pathol..

[87] Guo X.D., Ji J., Xue T.F., Sun Y.Q., Guo R.B., Cheng H., Sun X.L. (2020). FTY720 Exerts Anti-Glioma Effects by Regulating the Glioma Microenvironment Through Increased CXCR4 Internalization by Glioma-Associated Microglia. Front. Immunol..

[88] Chiba K., Adachi K. (2012). Discovery of fingolimod, the sphingosine 1-phosphate receptor modulator and its application for the therapy of multiple sclerosis. Future Med. Chem..

[89] Hawkins C.C., Ali T., Ramanadham S., Hjelmeland A.B. (2020). Sphingolipid Metabolism in Glioblastoma and Metastatic Brain Tumors: A Review of Sphingomyelinases and Sphingosine-1-Phosphate. Biomolecules.

