# Supplementary material for: Sphingosine-1-Phosphate Recruits Macrophages and Microglia and Induces a Pro-Tumorigenic Phenotype That Favors Glioma Progression
Source: Cancers (Basel). 2023 Jan 12;15(2):479. doi: 10.3390/cancers15020479 (PMC9856301; doi:10.3390/cancers15020479)
Supplement: Supplementary file 1 [file cancers-15-00479-s001.zip › cancers-2100484-supplementary.pdf]

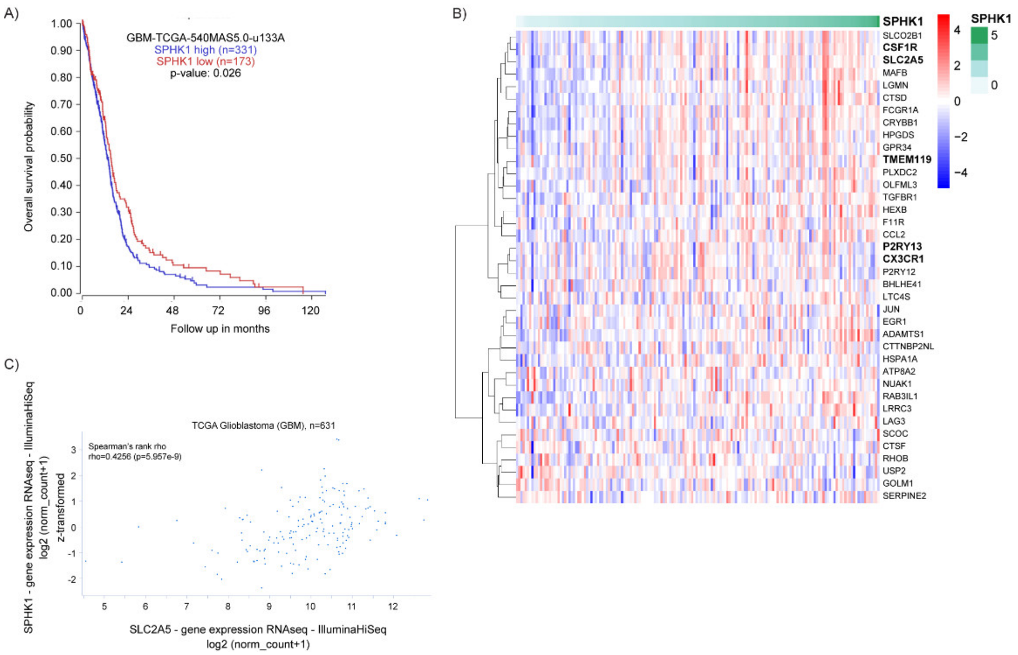


**Figure S1. High SPHK1 expression strongly correlates with higher microglia recruitment.** A) Kaplan-Meier estimates overall survival in patients with glioblastoma divided into high and low SPHK1 expression. Data analyzed using the Affymetrix 540 MASS 5.0-u133 array available in the online R2: Genomics Analysis and Visualization Platform (http://r2.amc.nl). B) Heatmap representation of microglia gene signature as reported in Butovsky et al., 2014 in TCGA RNAseq data of GB dataset and sorted according to SPHK1 expression level. C) Scatter plot showing the correlation between SPHK1 and SLC2A5 gene expression. Data analyzed using the RNAseq-IlluminaHiSeq GB data available in the online tool XENA from UCSC (https://xena.ucsc.edu/). p value in Kaplan Meier results from scan modus test.

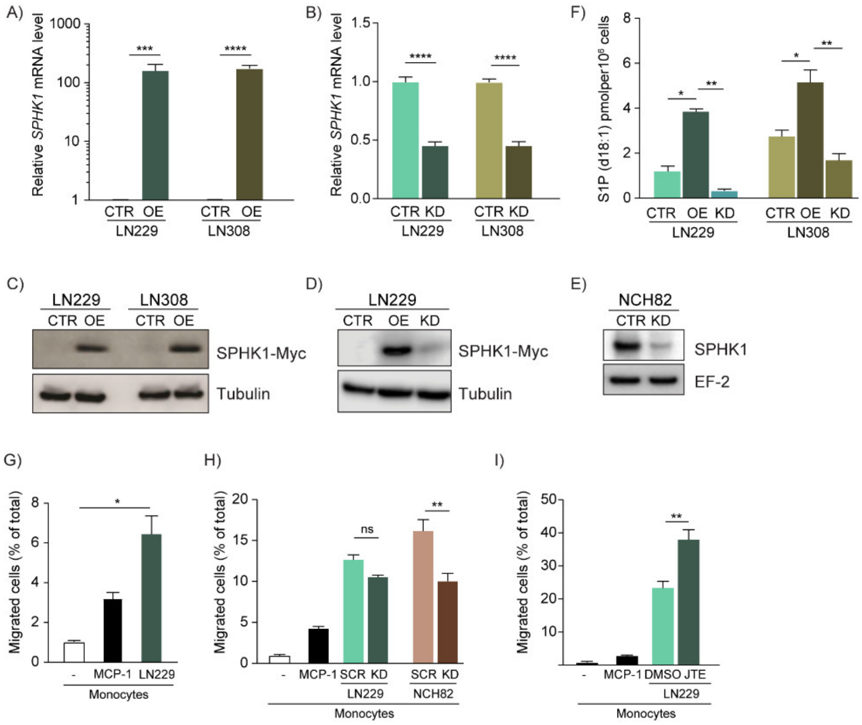


**Figure S2.** SPHK1 modulation is effective both at the expression and protein level and results in altered S1P secretion and higher monocytes chemoattraction. A, B) Quantitative RT-PCR analysis of SPHK1 in human glioma cells, LN229 and LN308, stably transfected with SPHK1-myc to induce SPHK1 overexpression (OE, A) or with shSPHK1 to promote the knockdown (KD, B). EGFP and scramble sh were used as control (CTR), respectively. The target gene was normalized to the mean of two housekeeping genes (PPIA, GAPDH). Data are expressed as fold-change over corresponding controls. C-E) Western blot analysis of SPHK1 expression in LN229, LN308 and NCH82 stably transfected with either SPHK1-myc (OE) or with shSPHK1 (KD) compared with respective controls (CTR). Tubulin or EF-2 served as loading controls. F) Quantification of S1P level by LC-MS/MS in LN229 and LN308 stably transfected with control, SPHK1-myc (OE) or with shSHK1 (KD) vectors. Data are expressed as S1P [d18:1] pmol per 106 cells. G, H) Chemoattraction assay of human monocytes towards LN229 and NCH82 either wild type (G) or knockdown for SPHK1 (H) versus corresponding control. I) Chemoattraction assay of human monocytes towards LN229 treated with JTE-013 or solvent control. MCP-1 was used as a positive control for chemoattraction. Data are expressed as percent increased or decreased migration in a time frame of 3 hrs. Mean with SEM are shown, n=3, *$p<0.05$, **$p<0.01$, ***$p<0.001$, ****$p<0.0001$ by Mann-Whitney test in A and B, Kruskal-Wallis test in C, G-I.

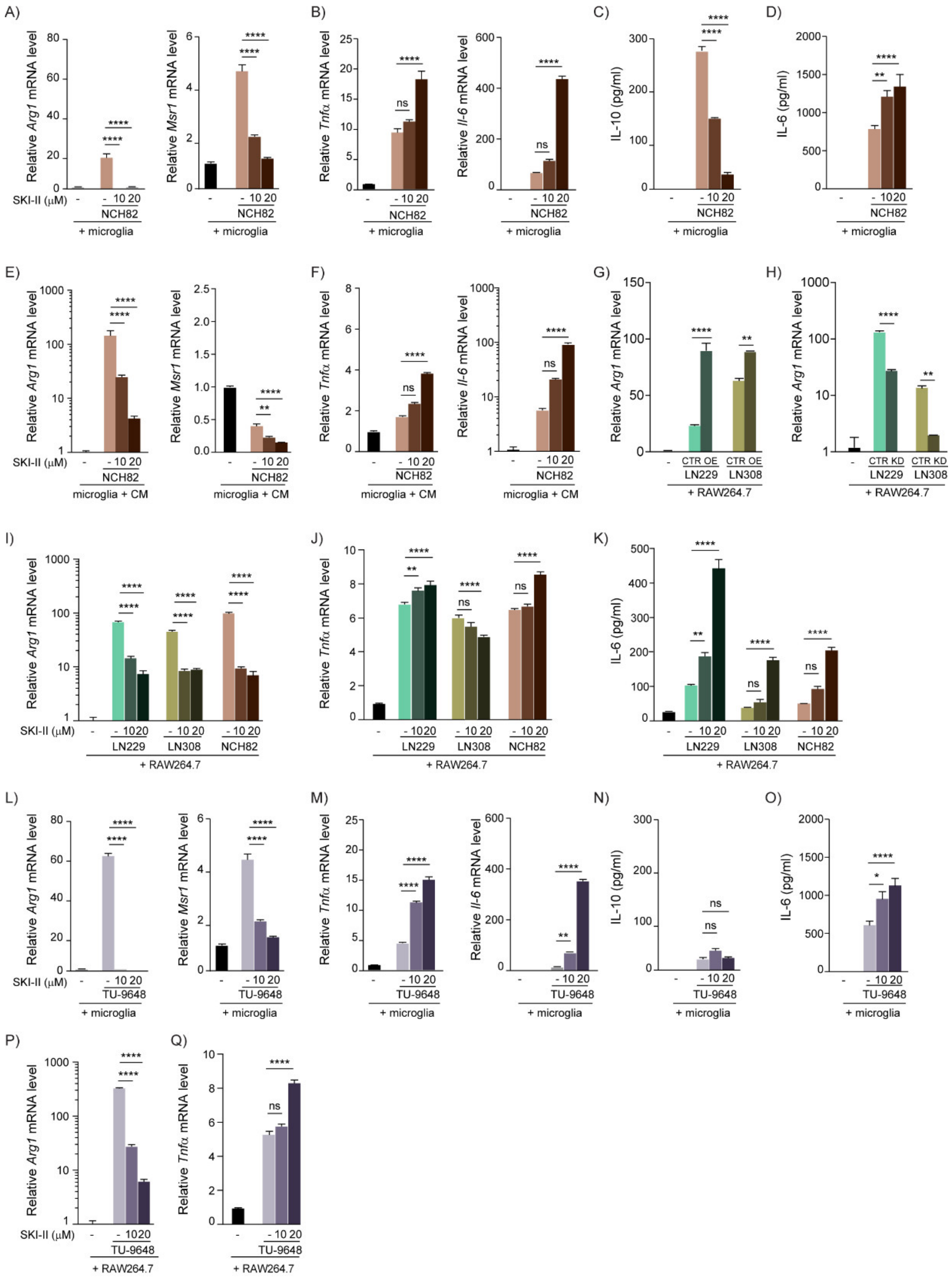


**Figure S3.** S1P shifts TAMs to an anti-inflammatory phenotype, whereas inhibition of SPHK1 activity reverts this phenotype into a pro-inflammatory one. A, B) Quantitative RT-PCR analysis of M2 markers Arg1 and Msr1 (A) and M1 markers Tnfα and Il-6 (B) in murine primary microglia co-cultured with human NCH82 glioma cells and treated for 24h with 10 or 20 μM of SKI-II or corresponding solvent control. C, D) ELISA of M2 cytokine IL-10 (C) and M1 cytokine IL-6 (D) in cell-free supernatant of murine primary microglia co-cultured with NCH82, upon treatment with SKI-II for 24 hrs. E, F) Quantitative RT-PCR analysis of Arg1 and Msr1 (E) and Tnfα and Il-6 (F) in murine microglia cultured in media conditioned by NCH82 (CM), upon treatment for 24 hrs with 10 or 20 μM of SKI-II or corresponding control. G, H) Quantitative RT-PCR analysis of Arg1 in murine RAW264.7 macrophages co-cultured with LN229 or LN308, overexpressing (OE, G) or knockdown (KD, H) for SPHK1. I, J) Quantitative RT-PCR analysis of Arg1 (I) and Tnfα (J) in RAW264.7 macrophages co-cultured with LN229, LN308 and NCH82, and treated for 24 hrs with 10 and 20 μM of SKI-II or corresponding control. K) ELISA of M1 cytokine IL-6 in cell-free supernatant of RAW264.7 macrophages co-cultured with LN229, LN308 and NCH82, upon treatment with SKI-II for 24 hrs. L, M) Quantitative RT-PCR analysis of Arg1 and Msr1 (L) and

Tnfα and Il-6 (M) in murine primary microglia co-cultured with murine TU-9648 glioma cells and treated for 24 hrs with 10 or 20 μM of SKI-II or corresponding solvent control. N, O) ELISA of IL-10 (N) and IL-6 (O) in cell-free supernatant of murine primary microglia co-cultured with TU-9648 cells, upon treatment with SKI-II for 24 hrs. P, Q) Quantitative RT-PCR analysis of Arg1 (P) and Tnfα (Q) in RAW264.7 macrophages co-cultured with TU-9648 cells, and treated for 24 hrs with 10 and 20 μM of SKI-II or corresponding control. In all the qPCR analysis, target genes were normalized to the mean of two housekeeping genes (B2m, Hprt). Data are expressed as fold-change over microglia or RAW264.7 cells cultured without glioma cells or conditioned medium. In all the ELISA, data are expressed as pg/ml as plotted against a standard curve for each cytokine. Mean with SEM are shown, n=3, ns: not significant, *p<0.05, **p<0.01, ***p<0.001, ****p<0.0001 by One-way ANOVA. .

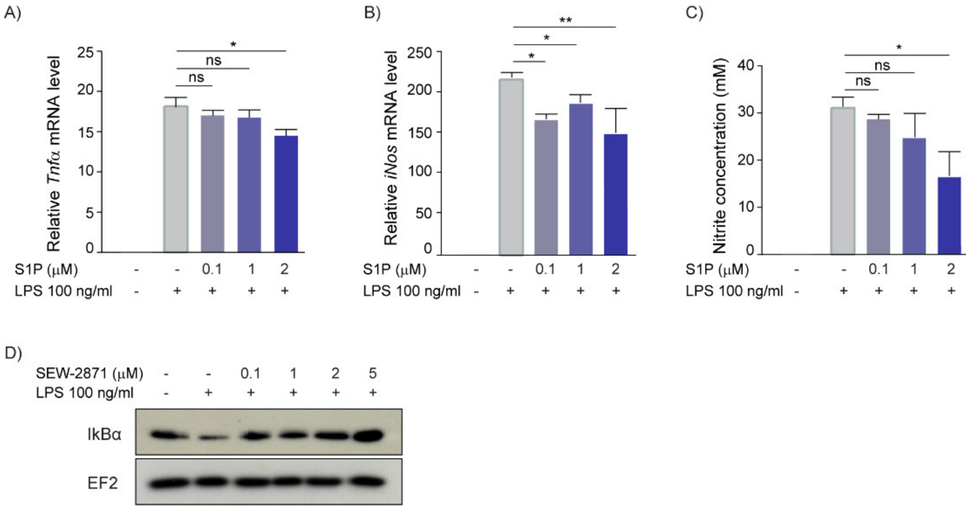


**Figure S4. S1P reduces LPS-induced inflammation.** A, B) Quantitative RT-PCR analysis of M1 markers Tnf*α* (A) and iNos (B) in RAW264.7 macrophages co-treated with LPS and increasing concentrations of S1P (0 – 2 μM) for 5 hrs. Target genes were normalized to the mean of two housekeeping genes (B2m, Hprt). Data are expressed as fold-change over cells treated with solvent control. C) Nitrite levels (NO2-) assessed by Griess reagent assay in RAW264.7 macrophages co-treated with LPS (100 ng/μL) and increasing concentration of S1P (0 – 2 μM) for 24 hrs. Mean with SEM are shown, n=3, ns: not significant, *p<0.05, **p<0.01, ***p<0.001 by Kruskal-Wallis test. D) Western blot analysis of IκB*α* amounts in murine primary microglia treated with LPS and increasing concentrations of S1P analogue SEW-2871 (0 – 5 μM, C) for 1 hr. EF2 served as loading control.

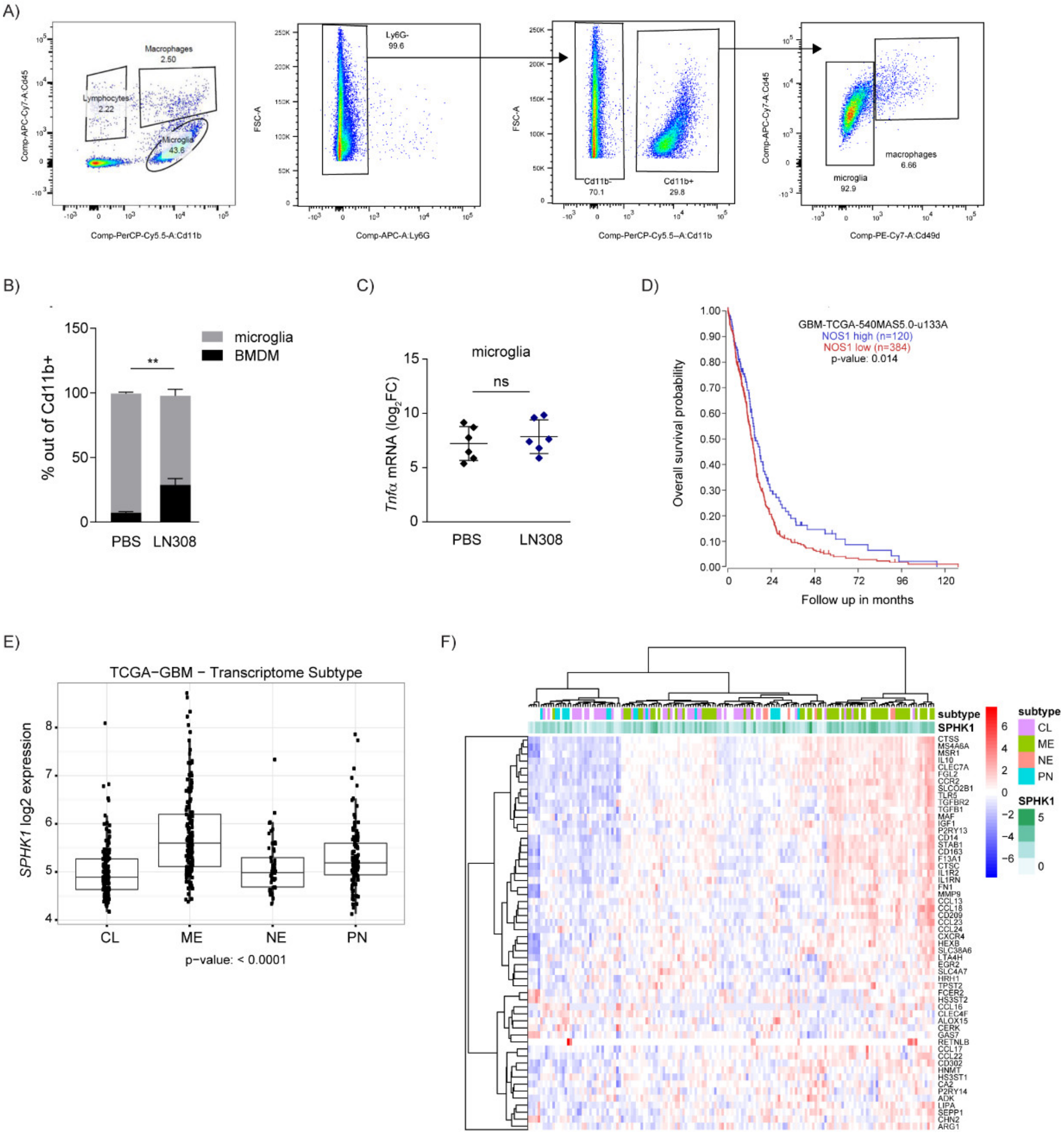

**Figure S5. Increased expression of pro-inflammatory genes correlates with a better survival in GB.** A) Gating strategy used to define microglia (Cd11b+/Cd45low/Cd49dlow) and BMDM (Cd11b+/Cd45high/Cd49dhigh) populations from dissociated mouse brains. B) The amount of microglia (Cd11b+/Cd45low/Cd49dlow) and BMDM (Cd11b+/Cd45high/Cd49dhigh) are quantified by FACS based on the abundance of the corresponding expression markers and expressed as percentage out of Cd11b+ cells. C) Quantitative RT-PCR analysis of M1 marker Tnfα in FACS-sorted microglia population. The target gene was normalized to the mean of two housekeeping genes (B2m, Hprt). Data are expressed as log2FC normalized to PBS mice. Mean with SD are shown, n=6, ns: not significant, **p<0.01 by unpaired t-test. D) Kaplan-Meier estimates overall survival in patients with GB divided into high and low NOS1 expression. Data analyzed using the Affymetrix 540 MASS 5.0-u133 array available in the online R2: Genomics Analysis and Visualization Platform (http://r2.amc.nl). E) SPHK1 expression measured in classical (CL), mesenchymal (ME), neural (NE) and proneural (PN) GB subtypes as in the TCGA Transcriptome GB dataset. F) Heatmap of M2 genes in TCGA Affimetrix GB dataset. Samples are clustered according to subtypes classification and SPHK1 expression level. p value in Kaplan Meier results from scan modus test.

A)

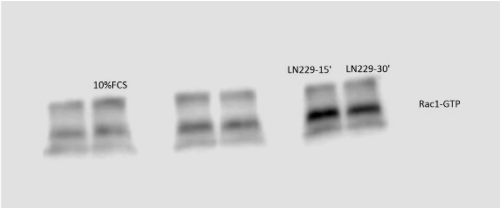


B)

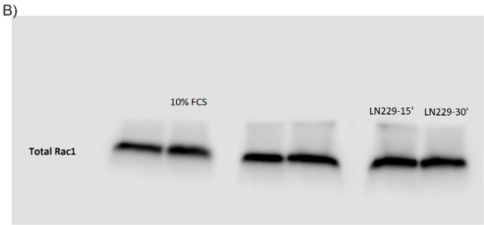


C)

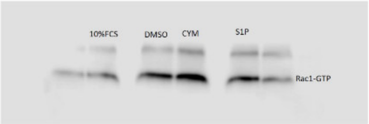


D)

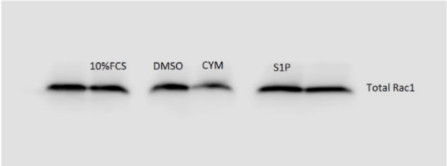


E)

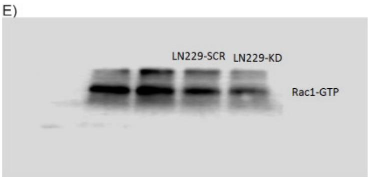


F)

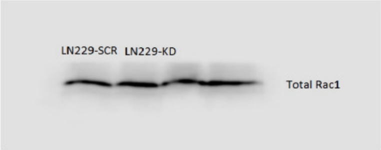


G)

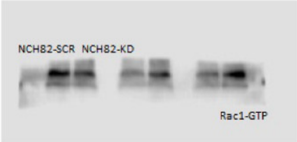


H)

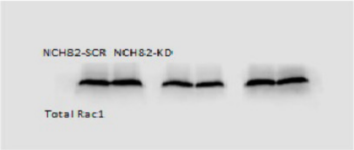


I)

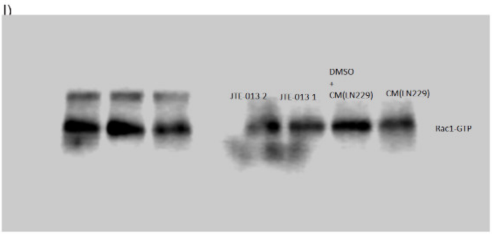


J)

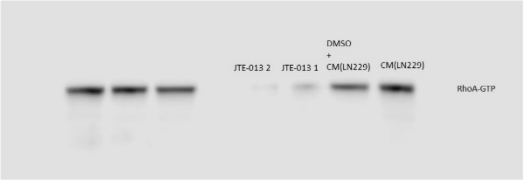


K)

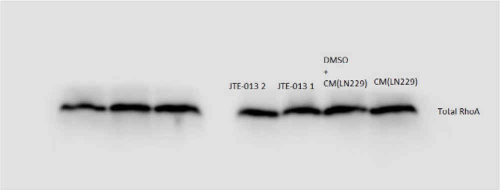


L)

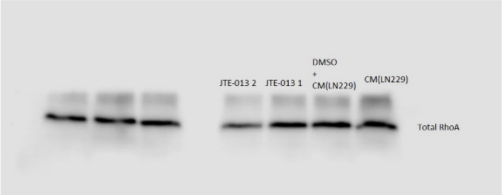


M)

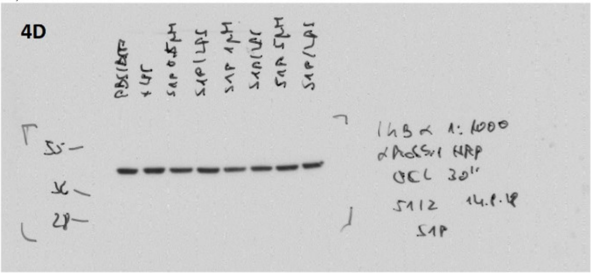

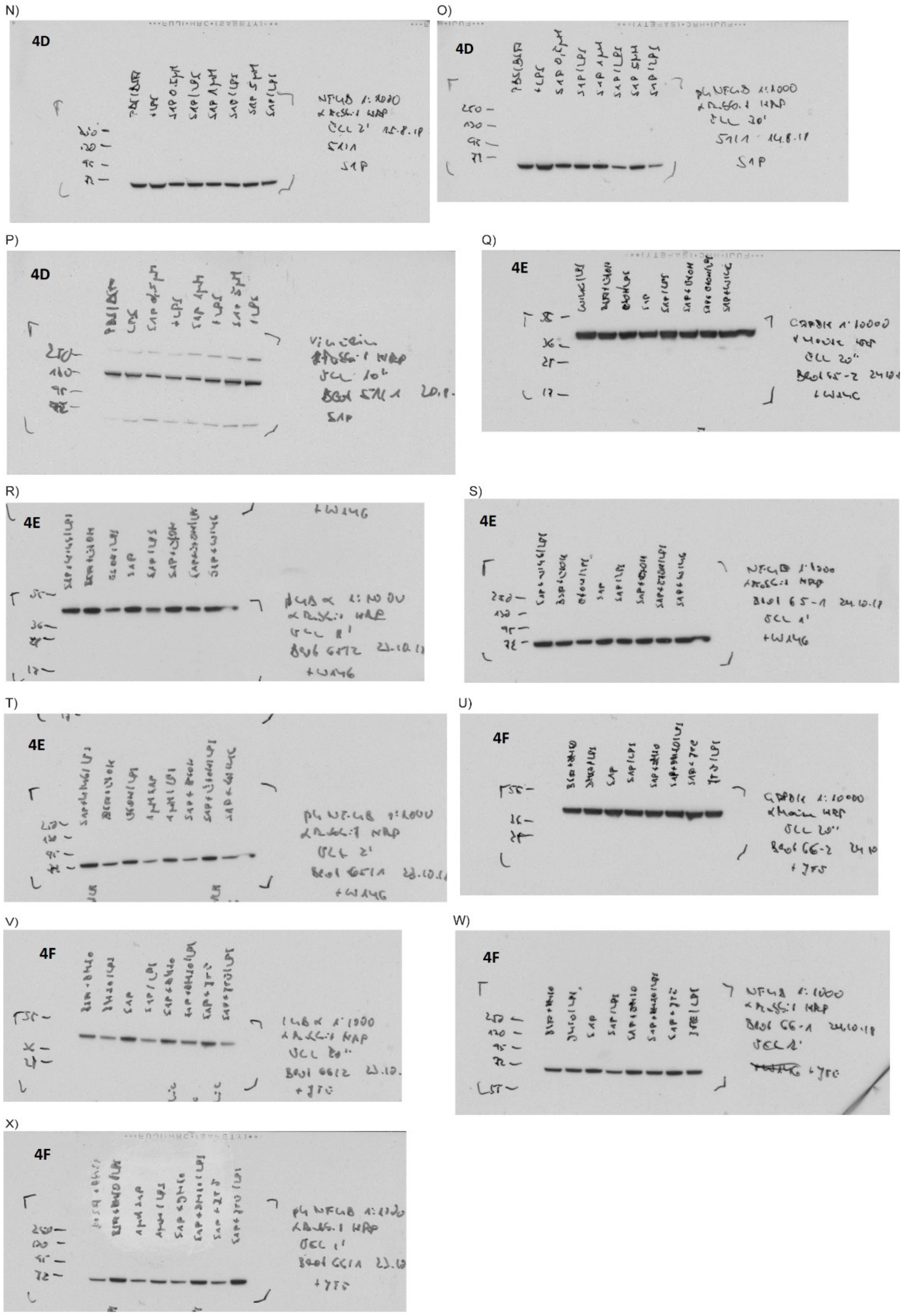


**Figure S6.** Original WB files.

## Supplementary Tables

Supplementary Table S1: Cell lines

| CELL LINE | SOURCE |
|---|---|
| HEK293 (RRID:CVCL_0045) | ATCC, USA |
| RAW264.7 (RRID:CVCL_0493) | ATCC, USA |
| LN229 (RRID:CVCL_0393) | ATCC, USA |
| LN308 (RRID:CVCL_0394) | ME Hegi (Lausanne University, Switzerland) |

Supplementary Table S2: Plasmids

| NAME | DESCRIPTION |
|---|---|
| pLKO.1-SPHK1 | shRNA against SPHK1 |
| pLKO.1-shCTR | Control shRNA |
| pLVX- SPHK1-myc | SPHK1with N-terminal myc-tag |
| pLVX-tRFP-2A-Luc | Control; CDS fusion of tRFP and FLuc with porcine teschovirus-1 2A |

Supplementary Table S3: Short hairpin RNA

| | NAME | TARGET SEQUENCE 5′-3′ |
|---|---|---|
| hSPHK1 overexpression | Sphk1shRNAHum-F | CCG GAC CTA GAG AGT GAG AAG TAT CCT CGA GGA TAC TTC TCA CTC TCT AGG TTT TTT TG |
| | Sphk1shRNAHum-R | CTA GCA AAA AAA CCT AGA GAG TGA GAA GTA TCC TCG AGG ATA CTT CTC ACT CTC TAG GT |
| hSPHK1 knockdown | hSPHK1-A-shRNA-F | CCG GCC TGA CCA ACT GCA CGC TAT TCT CGA GAA TAG CGT GCA GTT GGT CAG GTT TTT G |
| | hSPHK1-A-shRNA-R | AAT TCA AAA ACC TGA CCA ACT GCA CGC TAT TCT CGA GAA TAG CGT GCA GTT GGT CAG G |
| shCTR | control | CAACAAGATGAAGAGCACCAA |

Supplementary Table S4: Primers

| GENE NAME | PRIMER NAME | TARGET SEQUENCE 5′-3′ |
|---|---|---|
| **Human primers** | | |
| SPHK1 | SPHK1_F | GAG CAC CGG TGT CAT TCC |
| | SPHK1_R | CAG ACG TGG GCT GAG CTT |
| GAPDH | GAPDH_F | AGA AGG CTG GGG CTC ATT TG |
| | GAPDH_R | AGG GGC CAT CCA CAG TCT TC |
| PPIA | PPIA_F | TTC TGC TGT CTT TGG GAC CT |
| | PPIA_R | CAC CGT GTT CTT CGA CAT TG |
| | | |
| **Mouse primers** | | |
| B2m | B2m_F | TTC AGT ATG TTC GGC TTC CC |
| | B2m_R | TGG TGC TTG TCT CAC TGA CC |
| Gapdh | Gapdh_F | TTG ATG GCA ACA ATC TCC AC |
| | Gapdh_R | CGT CCC GTA GAC AAA ATG GT |
| ActB | ActB_F | ATG GAG GGG AAT ACA GCC C |

| | | |
|---|---|---|
| | ActB_R | TTC TTT GCA GCT CCT TCG TT |
| Hprt | Hprt1_F | CAT AAC CTG GTT CAT CAT CGC |
| | Hprt1_R | TCC TCC TCA GAC CGC TTT T |
| Arg1 | Arg1_F | GTG AAG AAC CCA CGG TCT GT |
| | Arg1_R | GCC AGA GAT GCT TCC AAC TG |
| Msr1 | Msr1_F | TTT CCC AAT TCA AAA GCT GA |
| | Msr1_R | CCT CCG TTC AGG AGA AGT TG |
| Il-6 | Il-6_F | TGG TAC TCC AGA AGA CCA GAG G |
| | Il-6_R | AAC GAT GAT GCA CTT GCA GA |
| iNos | iNos_F | TTC TGT GCT GTC CCA GTG AG |
| | iNos_R | TGA AGA AAA CCC CTT GTG CT |
| Il1b | Il1β_F | GGT CAA AGG TTT GGA AGC AG |
| | Il1β_R | TGT GAA ATG CCA CCT TTT GA |
| Tnfα | Tnfα_F | AGG GTC TGG GCC ATA GAA CT |
| | Tnfα_R | CCA CCA CGC TCT TCT GTC TAC |
| Cd206 | Cd206_F | GCAAATGGAGCCGTCTGTGC |
| | Cd206_R | CTCGTGGATCTCCGTGACAC |

Supplementary Table S5: Antibodies

| NAME | CLONE | CATALOGUE NO | MANUFACTURER |
|---|---|---|---|
| Cd11b | M1/70 | 45-0112-82 | eBioscience |
| Cd45 | 30-F11 | 103116 | Biolegend |
| Cd49d | R-2 | 103617 | Biolegend |
| CD14 | M5E2 | 301804 | Biolegend |
| EF2 | C-14 | sc-13004 | Santa Cruz Biotechnologies |
| GAPDH | 6C5 | CB1001-500UG | Merck Millipore |
| IkBα | | 9242 | Cell Signaling Technology |
| Ly6G | 1A8 | 17-9668-82 | eBioscience |
| NFκB-p65 | D14E12 | 8242S | Cell Signaling Technology |
| Phosphor-NFκB-p65 | 93H1 | 3033S | Cell Signaling Technology |
| Rac1 | | ARC03 | Cytoskeleton |
| RhoA | | ARH04 | Cytoskeleton |
| Sphk1 | | 71700 | Abcam |
| Tubulin | | T9026 | Sigma-Aldrich |
| Vinculin | N1N3 | gtx113294-100 | GeneTex |

Supplementary Table S6: Measurements of S1P derivatives analyzed by UPLC-ESI-(QqQ)-MS

| Time [min] | solvent A [%] | solvent B [%] |
|---|---|---|
| 0 | 70 | 30 |
| 1 | 70 | 30 |
| 2.5 | 30 | 70 |
| 4 | 20 | 80 |
| 5 | 20 | 80 |
| 6.5 | 10 | 90 |
| 6.6 | 0 | 100 |
| 7.5 | 0 | 100 |
| 7.6 | 70 | 30 |
| 9 | 70 | 30 |

Solvent A: Acetonitrile/water (1:4) + 0.1% formic acid
Solvent B: Acetonitrile/isopropanol (1:4) + 0.1% formic acid

Supplementary Table 7: Multiple reaction monitoring (MRM) for S1P measurements

| | Precursor Ion | Product Ion | |
|---|---|---|---|
| S1P(d17:1) (internal standard) (Rt = 2.70 min) | m/z 422 | m/z 60 | |
| | m/z 422 | m/z 113 | Quantifier |
| | m/z 422 | m/z 127 | |
| S1P(d18:1) (Rt = 2.89 min) | m/z 436 | m/z 60 | |
| | m/z 436 | m/z 113 | Quantifier |
| | m/z 436 | m/z 127 | |
